# Exosome-delivered bioactive molecules regulate macrophage polarization in atherosclerosis and myocardial infarction: mechanisms and therapeutic potential

**DOI:** 10.3389/fcvm.2026.1739907

**Published:** 2026-02-19

**Authors:** Yuanlin Zhou, Guanghe Ran, Hua Guo

**Affiliations:** 1 Department of Cardiology Dandong Central Hospital, Liaoning, China; 2 Chongqing Changshou Traditional Chinese Medicine Hospital, Chongqing, China

**Keywords:** atherosclerosis, exosome, macrophage, myocardial infarction, polarization

## Abstract

Exosomes, by carrying biologically active molecules, constitute the core network of inter-cell communication and play an important role in the regulation of macrophage polarization. The dynamic balance of macrophage polarization is a key determinant of atherosclerosis plaque stability and cardiac repair after myocardial infarction. This review systematically summarizes the molecular mechanisms by which exosomes and their specific molecules accurately regulate M1/M2 polarization of macrophages. We also focused on the mechanism of action by which exosomes play a dual role in promoting or inhibiting the physiological and pathological environment of AS and MI. In addition, the clinical transformation potential and current challenges of new biomarkers and treatment strategies (such as engineered exosomes, drug carriers) are also discussed, which is expected to bring new treatment strategies to the treatment of cardiovascular diseases.

## 1 Introduction

Cardiovascular diseases (CVDs), particularly atherosclerosis (AS) and myocardial infarction (MI), pose the foremost threat to global health. In recent years, the research focus on their pathogenesis has shifted from traditional metabolic abnormalities to chronic immune-inflammatory responses. This response underpins the entire continuum of AS, from its initiation and progression to plaque rupture and subsequent cardiac remodeling following MI, serving as a common pathological basis for disease evolution (1).

In this complex process, macrophages, serving as the core effector cells, exhibit remarkable plasticity. They can polarize into distinct functional phenotypes in response to microenvironmental signals. Early research oversimplified this paradigm into a dichotomy: pro-inflammatory M1 (classically activated) and anti-inflammatory/reparative M2 (alternatively activated) phenotypes (2). However, current understanding has moved beyond the classical M1/M2 dichotomy, revealing a continuous spectrum of functional states. In AS, the macrophage phenotype shifts from an early, predominantly lipid-clearing, and anti-inflammatory state to a late, pro-inflammatory state that drives inflammation and compromises plaque stability. Conversely, in MI, their function transitions from early inflammatory clearance to repair and reconstruction; dysregulation of this transition directly contributes to adverse remodeling and heart failure (3). Consequently, the precise modulation of macrophage polarization represents a crucial therapeutic target for CVDs.

Efficient intercellular communication is the foundation for coordinating the aforementioned processes. Exosomes—nanoscale vesicles actively secreted by cells—serve as a central medium in this communication. Acting as “Trojan horses” between cells, they can stably deliver functional signals over long distances, thereby reprogramming the fate of recipient cells (4–6). Its function stems from the diverse “cargo” it carries, including proteins, lipids and nucleic acids (such as key miRNAs), whose composition accurately reflects the physiological and pathological state of the parent cell (7, 8). Therefore, exosomes become natural nanotools for regulating macrophage polarization.

Based on the above background, this review aims to deeply and systematically explore the specific molecular mechanisms and functional effects of the “exosome-macrophage” axial dialogue in atherosclerosis and myocardial infarction. First, we will elaborate on how exosomes regulate the recruitment, polarization and function of macrophages through the specific miRNAs, proteins and other active molecules they carry, thereby affecting the fate of AS plaques (stability vs. rupture) and heart repair after MI. Process (functional repair vs. poor fibrotic remodeling). Finally, we will critically evaluate the current research status of exosomes as therapeutic agents or drug carriers, analyze the challenges they face on the road to clinical transformation (such as standardized production, targeted delivery efficiency, safety), and look forward to this A promising field. The future direction of development.

## 2 Exosome biology: biogenesis, function

**Figure 1:**
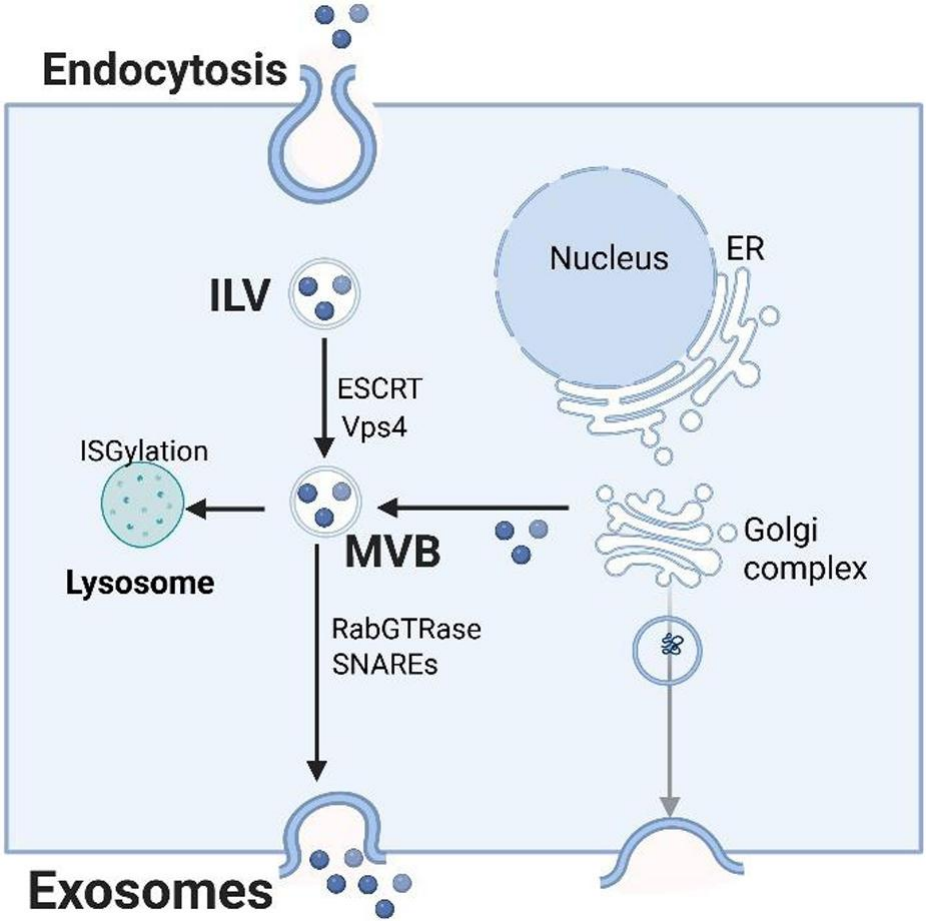
Exosome biogenesis, sorting, and trafficking.

The biogenesis of exosomes is closely regulated by the body (Figure 1). It originates from the endosomal system: invaginates of the cell membrane forms early endosomes [intraluminal vesicles (ILVs)], which in turn develop into multimular bodies (MVB). The process relies on the endosomal sorting complex required for transport (ESCRT) (9), which includes multiple components such as ESCRT-0, ESCRT-I, ESCRT-II, ESCRT-III, and Vps4 (10, 11). ESCRT-0 is mainly responsible for aggregating ubiquitinated proteins, ESCRT-I/II assists membrane bending, ESCRT-III drives membrane shearing, and Vps4 promotes the depolymerization and recovery of the complex. Interestingly, ILV can still form MVB when ESCRT expression is suppressed, indicating the existence of a non-ESCRT pathway (12). For example, neutral sphingomyelin 2 (nSMase2) promotes ILV invaginates and MVB formation by producing ceramides, a process that can be blocked by inhibitors such as GW4869 (13). After MVB is formed, it can be degraded by lysosomes, or it can fuse with the cell membrane through SNAREs and RabGTPase to release exosomes in the form of exocytosis. In addition, transmembrane proteins (such as CD9, CD81, heat shock proteins, TSG101, etc.) and integrin proteins (such as ITGA3, ITGB1) play an important role in the production of exosomes and participate in regulating cell homeostasis (4, 14–17).

Exosomes carry a variety of biologically active substances such as proteins, lipids, and nucleic acids. They participate in and regulate a variety of physiological and pathological processes such as cancer development, immune response, and cardiovascular disease through two core mechanisms: First, their surface molecules directly interact with target cells. Receptor binding activates signaling pathways, second, fusion with target cell membranes to deliver contents to change cell functions. Different cell types and microenvironments can regulate the production mechanism and components of exosomes, showing obvious spatiotemporal and cell specificity.

## 3 Signaling mechanisms of exosome-mediated macrophage polarization

**Figure 2:**
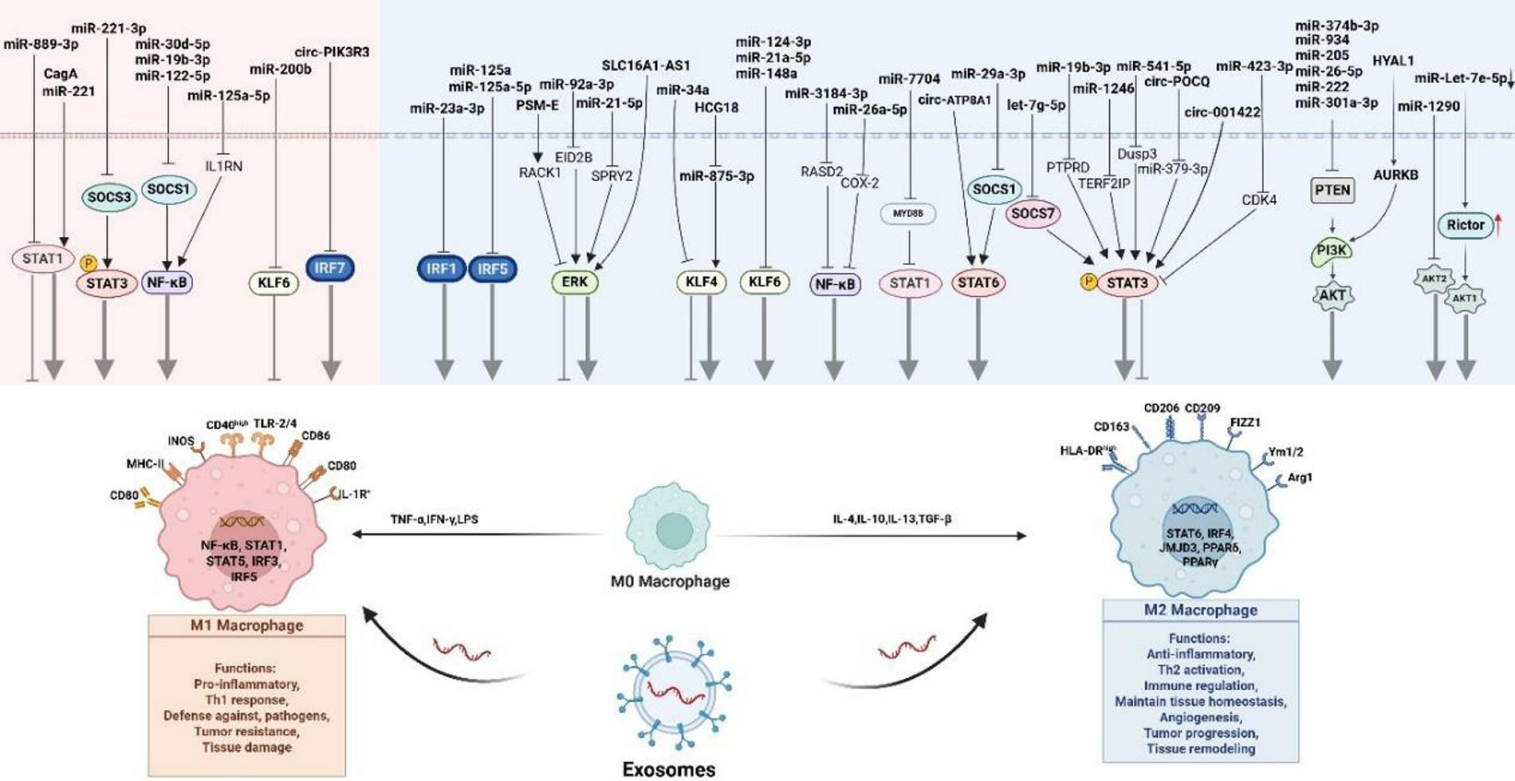
The mechanism of exosomes mediating macrophage polarization. Macrophages can be polarized into M1 and M2 types under different conditions and play different roles. Exosomes can mediate macrophage polarization through key signaling pathways such as PI3K/AKT, STAT, NF-*κ*B, Krippel-like transcription factors, extracellular signal-regulated kinases (ERK), and interferon regulatory factors (IRF).

The plastic polarization of macrophages plays a central role in the regulation of disease process. Macrophages can be stimulated to polarize into M1 and M2 types under different environmental conditions. In view of the many reviews on the polarization mechanism of macrophages in recent years, we will not elaborate on it here. Please refer to it for details (18, 19). Exosomes, as key carriers of inter-cell communication, have been shown to reprogram macrophage phenotypes by delivering multiple active molecules. This process involves a complex signaling network, among which key signaling pathways such as PI3 K/AKT, STAT, NF-*κ*B, Krippel-like transcription factors (KLF), extracellular signal-regulated kinase (ERK), and interferon regulatory factor (IRF) constitute the core regulator*y* axis. Below we summarize the latest molecular mechanisms by which exosomes mediate macrophage polarization through the above signaling pathways in recent years (Figure 2), providing new ideas and targets for immunotherapy of related diseases (especially cardiovascular diseases).

### 3.1 PI3K/AKT and phosphatase and tensin homolog (PTEN)

A major mechanism of PTEN-mediated inhibition has been attributed to its function as an inhibitor of the PI3 K/AKT pathway (20). It has been reported in the literature that exosomes can affect the PI3 K/AKT signaling pathway through PTEN, further affecting macrophage polarization. Huang et al. discovered a new mechanism for promoting cancer by exosome miR-205: after it is phagocytized by macrophages, it targets PTEN, activates the PI3 K/AKT/mTOR signal axis, drives the M2 polarization of macrophages, and ultimately accelerates ovarian cancer metastasis (21). seminal plasma derived exosomes are rich in miR-26-5p, which can be internalized by uterine macrophages. By down-regulating PTEN, they activate the PI3 K/AKT signaling pathway and drive macrophages to polarization towards the M2 phenotype, which is conducive to pregnancy maintenance (22). In colorectal cancer, particularly with liver metastasis, elevated exosomal miR-934 is a marker of poor prognosis. Research demonstrates that cancer-derived exosomal miR-934 promotes M2 macrophage polarization via PTEN suppression and PI3 K/AKT activation. These polarized macrophages further secrete CXCL13, and the ensuing CXCL13-CXCR5 axis drives the colonization of liver by metastatic cancer cells (23). Furthermore, other exosomal miRNAs, including miR-222 (24) and miR-301a-3p (25, 26), have been reported to operate through this conserved mechanism of targeting PTEN to activate PI3 K/AKT signaling and drive macrophage polarization.

Other exosomes can also mediate macrophage polarization through this signaling pathway. Glioblastoma stem cells deliver highly expressed miR-374b-3p to macrophages through exosomes, and induce their M2 polarization by down-regulating PTEN, thereby promoting tumor angiogenesis and accelerating disease progression (27). In a study on brucellosis, Li et al. discovered that serum exosomes from patients exhibit significantly downregulated miR-let-7e-5p. This reduction attenuates its inhibitory effect on the target gene Rictor in macrophages, leading to hyperactivation of the AKT1 signaling pathway and driving macrophages toward an M2 phenotype. The resulting immunosuppressive microenvironment facilitates intracellular survival of Brucella and disease progression. These findings not only elucidate a precise mechanism of exosomal miRNA-mediated immunomodulation but also suggest the miR-let-7e-5p/Rictor axis as a potential therapeutic target for brucellosis (28). Furthermore, exosomes derived from esophageal squamous cell carcinoma are enriched with hyaluronidase 1 (HYAL1). Upon uptake by macrophages, exosomal HYAL1 interacts with and activates the AURKB/PI3 K/AKT signaling cascade, promoting M2 polarization. This phenotypic shift remodels the tumor microenvironment and fuels ESCC malignancy (29). Hypoxic hepatocellular carcinoma cells deliver highly expressed miR-1290 to macrophages via exosomes. miR-1290 drives macrophages to M2 phenotype polarization by inhibiting Akt2 and upregulating PD-L1, which in turn induces apoptosis of CD8 ^+^ T cells and promotes epithelial-mesenchymal transition (EMT), ultimately accelerating tumor immune escape and metastasis (30).

To sum up, exosomes (carrying miRNAs, proteins, etc.) activate the PI3K/AKT signaling pathway by regulating upstream molecules such as PTEN, which is a highly conserved core mechanism that induces M2 polarization in macrophages. This mechanism is widely found in a variety of diseases and highlights its potential as an important the treatment target by reshaping the disease process in the immune microenvironment.

### 3.2 STAT

The STAT family plays an important role in macrophage polarization, especially STAT3. The exosomal miR-1246 from hypoxic gliomas is a key regulator that induces M2 macrophage polarization, subsequently accelerating tumor progression *in vitro* and *in vivo*. This effect is mediated through the targeting of TERF2IP by miR-1246 to activate STAT3 signaling and suppress NF-*κ*B signaling (31). miR-221, the exosome of breast epithelial cells, inhibits the expression of SOCS1 and the phosphorylation of STAT3. By promoting STAT1 phosphorylation, it activates STAT1-mediated polarization of M1 macrophages and promotes the inflammatory response (32). The exosome of lung adenocarcinoma origin, miR-19b-3p, mediates the dephosphorylation of STAT3 in macrophages by targeted inhibition of PTPRD, leading to STAT3 activation and inducing M2 polarization in macrophages. Exosome miR-19b-3p promotes the polarization of M2 macrophages and also promotes the secretion of macrophage exosome LINC00273. LINC00273 promotes lung adenocarcinoma metastasis through interaction with the Hippo pathway of tumor cells (33). Notably, miR-423-3p is downregulated in the plasma exosomes of cervical cancer patients, implying its role as a potential suppressor of cancer progression. According to Yan et al., this miRNA modulates M2 macrophage polarization by directly targeting and inhibiting CDK4. The subsequent decrease in CDK4 levels suppresses STAT3 phosphorylation, which in turn diminishes IL-6 secretion and ultimately reduces M2 macrophage polarization (34). Cigarette smoke exposure induces human bronchial epithelial cells to secrete miR-221-3p-rich exosomes. After the exosomes are taken up by macrophages, they activate the STAT3 signaling pathway by targeted inhibition of SOCS3, driving macrophages to polarize towards the M1 phenotype, thereby exacerbating the inflammatory process in chronic obstructive pulmonary disease (35). Gastric cancer cells deliver miR-541-5p to macrophages through exosomes. By targeting inhibition of DUSP3, it removes its inhibitory effect on the JAK2/STAT3 signaling pathway, thereby driving macrophages to polarize towards the M2 phenotype and promoting tumor malignant progression (36). Cao et al. revealed that IL-6 induces the expression of circ-001422 in glioma cells. This circular RNA is subsequently packaged into exosomes and delivered to macrophages. Within macrophages, circ-001422 recruits p300 to promote STAT3 acetylation, thereby activating the STAT3 signaling pathway. This cascade ultimately drives M2 macrophage polarization and accelerates glioma progression (37). A recent study identified SERPINE1 as a critical bifunctional regulator in gastric cancer. Intracellularly, it activates the JAK2/STAT3 pathway to promote let-7g-5p transcription. Let-7g-5p is then shuttled via exosomes to macrophages, where it targets inhibition of SOCS7, relieving its inhibition of STAT3 and thereby driving M2 polarization (38). Similarly, exosomes from colorectal cancer cells transport highly expressed circPOLQ to macrophages. Within macrophages, circPOLQ functions as a competitive endogenous RNA that sequesters miR-379-3p. This sequestration relieves the suppression of miR-379-3p target genes, thereby activating the IL-10/STAT3 signaling axis. This cascade promotes M2 polarization and ultimately facilitates the formation of metastatic nodules in CRC (39).

Naturally, STAT1 and STAT6 in the STAT family are also crucial in macrophage polarization. Lin et al. identified the exosome miR-7704 as a key molecule driving the polarization of macrophages towards the M2 phenotype. It acts by inhibiting the MyD88/STAT1 signaling pathway. In the acute lung injury (ALI) model, direct delivery of miR-7704 can effectively induce M2 polarization of macrophages in the lungs, repair the alveolar barrier, improve lung function and improve survival, demonstrating its great potential as a new the therapies drug (40). Similarly, cancer-associated fibroblasts (CAFs) deliver miR-889- 3p to macrophages via exosomes. By directly targeting inhibiting STAT1 expression, this miRNA blocks the polarization of macrophages towards the anti-tumor M1 phenotype, thereby creating an immunosuppressive microenvironment and ultimately promoting the malignant progression of esophageal squamous cell carcinoma (41). CagA, the virulence factor of Helicobacter pylori, can be delivered to macrophages by exosomes of gastric epithelial cells. By activating the JAK/STAT1 signaling pathway, on the one hand, it induces the polarization of macrophage M1, and on the other hand, it promotes STAT1 entry into the nucleus and down-regulates the expression of SLC7A11, induces iron death, and jointly exacerbates gastric mucosal inflammation (42). Gastric cancer cells secrete exosomes carrying highly expressed circATP8A1, which are taken up by macrophages. Inside macrophages, circATP8A1 functions as a molecular sponge for miR-1-3p, sequestering it and thus relieving its suppression of STAT6. This specific activation of the STAT6 (as opposed to STAT3) pathway drives M2 polarization and ultimately accelerates gastric cancer progression (43). In oral squamous cell carcinoma, tumor-derived exosomal miR-29a-3p promotes M2 polarization by targeting and inhibiting the negative regulator SOCS1 in macrophages, thereby upregulating STAT6 signaling (44).

### 3.3 NF-*κ*B

Exosomal miRNAs can also act on NF-*κ*B and affect the polarization balance of macrophages. Research by Jiao et al. demonstrates that in sepsis-related ALI, neutrophil exosomes deliver miR-30d-5p to macrophages. This miRNA promotes M1 polarization and lung injury progression by targeting SOCS-1 and SIRT1 to activate NF-*κ*B signaling. The finding that a miR-30d-5p inhibitor alleviates the condition by reducing M1 macrophages underscores a new intercellular communication pathway (45). Exosomal miR-19b-3p from renal tubular epithelial cells is taken up by macrophages and drives their M1 polarization. This is achieved by targeting SOCS-1 to relieve its inhibition on the NF-*κ*B signaling pathway, thereby activating this pro-inflammator*y* axis (46). In glioma, miR-3184-3p promotes tumorigenesis through direct effects on cancer cells and indirect modulation of the microenvironment: it enhances tumor cell proliferation, migration, and resistance to apoptosis while also inhibiting RASD2 in macrophages to suppress NF-*κ*B signaling, thereby inducing M2 polarization and fostering an immunosuppressive milieu that accelerates tumor growth (47). Separately, in the context of liver transplantation, Lyu et al. identified that ischemia-reperfusion injury induces the release of exosomal miR-122-5p, which, upon delivery to alveolar macrophages, targets SOCS1 to activate NF-*κ*B signaling. This promotes M1 polarization and contributes to ALI, a process supported by clinical observations linking exosomal miR-122-5p levels to disease severity in children (48). Cigarette smoke exposure induces bronchial epithelial cells to secrete exosomes carrying miR-125a-5p. This miRNA, upon delivery to macrophages, targets and inhibits IL1RN, leading to activation of the MyD88/NF-*κ*B pathway and driving pro-inflammatory M1 polarization, which potentiates lung inflammation in COPD. This mechanism unveils a new potential target for intervening in COPD progression (49). The efficacy of Qingrehuoxue Decoction (QRHX) stems in part from its regulation of exosomes of bone marrow-derived macrophages. QRHX promotes exosomes to enrich miR-26a-5p. After this miRNA is delivered to macrophages in the lesion area, it directly targets and inhibits PTGS2 (COX-2), which in turn blocks the NF-*κ*B signaling pathway and drives macrophages to transform from a pro-inflammatory M1 to an anti-inflammatory M2 phenotype, thereby playing an anti-inflammatory and plaque stabilizing role (50).

To sum up, exosomal miRNAs affect macrophage polarization in both directions by regulating the NF-*κ*B signaling pathway: on the one hand, they activate NF-*κ*B to drive the pro-inflammatory M1 phenotype to aggravate inflammatory diseases, on the other hand, they inhibit this pathway to induce the anti-inflammatory M2 phenotype to promote tumor progression. This mechanism provides a new target for immunotherapy of related diseases.

### 3.4 Kruppel-like factor (KLF) family

The KLF family is involved in various aspects of cell growth, development, and differentiation. Accumulating evidence indicates that KLF transcription factors play regulatory roles in macrophage polarization. For instance, KLF4 promotes M1 polarization in rheumatoid arthritis by modulating STAT1 (51), while KLF14 suppresses M2 polarization by inhibiting STAT3 signaling activation (52).

Studies have found that the exosome miR-34a secreted by adipocytes inhibits M2 polarization by inhibiting the expression of KLF4 and promotes fat inflammation caused by obesity (53). Gastric cancer cells deliver highly expressed lncRNA HCG18 to macrophages through exosomes. In macrophages, HCG18 acts as a competitive endogenous RNA to adsorb miR-875- 3p, relieving its inhibitory effect on the key transcription factor KLF4, thereby driving macrophages to polarize towards the M2 phenotype, ultimately promoting the progression of gastric cancer. This mechanism has been confirmed *in vivo* experiments (54).

In ovarian cancer, highly expressed exosomal miR-200b from patient plasma targets KLF6 to inhibit M1 macrophage polarization, as evidenced by reduced levels of M1 markers (IL-1β, CD86). This alteration in the macrophage phenotype subsequently creates a microenvironment that fosters tumor cell proliferation and invasion (55). The exosomal miR-21a-5p from mesenchymal stem cells alleviates atherosclerosis by targeting KLF6. The downregulation of KLF6 promotes a shift in macrophages toward the M2 phenotype (indicated by elevated Arg-1), reduces overall macrophage infiltration, and thereby curbs disease development (56). Similarly, by downregulating KLF6, exosomal miR-148a promotes a phenotypic shift in macrophages from M1 to M2. This is characterized by the downregulation of pro-inflammatory markers (iNOS, IL-6, TNF-α) and the upregulation of anti-inflammatory markers (IL-10, CD206, Arg-1), ultimately attenuating inflammation (57). In a mouse model of ALI after lung transplantation, the researchers found that plasma exosome miR-124- 3p expression was significantly down-regulated. This miRNA inhibits the NF-*κ*B signaling pathway by targeting KLF6 in macrophages, thereby driving the transformation of macrophages to the anti-inflammatory and restorative M2 phenotype (58).

### 3.5 ERK (extracellular signal-regulated kinase)

ERK (Extracellular Signal-Regulated Kinase) is a core member of the MAPK signaling family and plays a crucial “decision-maker” role in the function of macrophages. Its core role is to integrate external signals and regulate the activation type (polarization) of macrophages, thereby determining their function in the immune response.

Zhao et al. found that the progression of colorectal cancer (CRC) is highly dependent on the polarization of M2-type macrophages in the tumor microenvironment (TME). Studies have revealed that CRC-derived exosomes target and inhibit the EID2B gene in macrophages by delivering highly expressed miR-92a-3p, which in turn activates the MAPK/ERK signaling pathway, thereby driving macrophages to polarization towards the M2 phenotype (59). Interestingly, hepatocellular carcinoma exosomes deliver lncRNASLC16A1-AS1 to macrophages, accelerating lactate influx by enhancing the stability of the lactate transporter SLC16A1, activating the c-Raf/ERK signaling pathway and inducing M2 polarization. Polarized M2 macrophages secrete IL-6, which feedbacks promote SLC16A1-AS1 expression in HCC cells, forming a positive feedback loop that drives tumor progression. This discovery clarifies a new mechanism by which exosome lncRNA regulates the tumor microenvironment through the metabolism-immune axis (lactate-ERK), providing a new target for HCC treatment (60). Exosomes derived from human umbilical cord mesenchymal stem cells can deliver rich miR-21- 5p to macrophages when injected into the mesenteric. By targeting SPRY2, miR-21- 5p activates the ERK signaling pathway, and drives macrophages to polarize towards the M2 phenotype, thereby alleviating mesenteric inflammation and colitis. This discovery clarifies the molecular mechanism by which HucMSCs-Exos regulates the immune microenvironment through the miR-21- 5p/SPRY2/ERK axis, providing a theoretical basis for its therapeutic application (61). Qin et al. found that PCa-derived exosomes are rich in proteasome activating subunit PSM-E, which recruits RACK1 protein to inhibit the activation of FAK and ERK signaling pathways, effectively preventing macrophages from polarizing toward the M2 phenotype (62).

In summary, in the tumor environment, exosomes activate the ERK pathway to promote M2 polarization and accelerate tumor progression; while in inflammatory diseases, activation of the ERK pathway induces M2 polarization to exert anti-inflammatory effects. This mechanism provides a new target direction for disease treatment.

### 3.6 IRF

Interferon regulatory factors (IRFs), a class of transcription factors initially identified as regulators of type I interferon gene expression, play pivotal roles in modulating pathogen-induced immune responses (63). Studies have shown that IRF transcription factors mediate the polarization of macrophages towards M2.

In a mouse model of tendon-bone healing following anterior cruciate ligament reconstruction (ACLR), bone marrow stromal cell-derived exosomal miR-23a-3p facilitated the transition of macrophages from the M1 to the M2 phenotype. This shift was characterized by a marked increase in anti-inflammatory factors such as IL-10 and TGF-*β*. Mechanistically, miR-23a-3p targets and suppresses IRF1 expression to mediate this phenotypic switch, thereby mitigating the early inflammatory response at the tendon-bone interface and promoting the initial healing process after ACLR (64). Furthermore, in spinal cord injury, exosomal miR-125a from bone marrow mesenchymal stem cells was found to reprogram macrophages toward the M2 phenotype, as indicated by elevated markers Arg1 and Ym1. This reprogramming occurs through the direct targeting and downregulation of the transcription factor IRF5, a mechanism that alleviates inflammation and provides neuroprotection (65). Exosomes are rich in miR-125a-5p and can be delivered to cardiac macrophages. By inhibiting the TRAF6/IRF5 signaling pathway, they drive them to polarize toward a repairable M2 phenotype, thereby significantly improving cardiac function and structural reconstruction after AMI. This strategy provides a new idea to enhance the efficacy of exosome-based cell-free therapy (66). Radiotherapy causes tumor cells to release exosomes rich in circPIK3R3. After the exosomes are taken up by macrophages, they upregulate IRF7 by adsorbing miR-872-3p through sponges, thereby driving macrophages to undergo M1 polarization and secrete type I interferon (I-IFN). I-IFN then activates the JAK/STAT pathway in CD8^+^T cells, enhancing their ability to produce IFN-*γ* and granzyme B, thereby effectively killing distant tumor cells (67). Interestingly, in ALI, adipose-derived stem cell exosomes (ADSC-Exos) mediate therapeutic effects through alveolar macrophages by downregulating IRF7. This downregulation achieves dual anti-inflammatory outcomes: inhibition of NLRP3 inflammasome-mediated pyroptosis and promotion of macrophage polarization from M1 to M2, collectively mitigating lung tissue damage (68).

### 3.7 Cross-dialogue in signal paths

A variety of biologically active substances carried by exosomes can activate multiple intracellular signaling pathways simultaneously or in a time-series manner. The extensive “cross-dialogue” between these pathways together constitutes a dynamic regulatory network that ultimately determines the polarization of macrophages. fate. The PI3 K/AKT-mTOR axis is at the core integration position in this network. It is not only an important transit station for growth factor and cytokine signaling, but also closely connects cellular energy status with immune response by regulating downstream metabolic and inflammation-related factors. For example, AKT can promote the activation of the NF-*κ*B pathway by activating IKK, while mTORC1 can also regulate the translation of I*κ*B*α*, bidirectionally regulating NF-*κ*B activity (69, 70). At the same time, the post-transcriptional modification of STAT3 by mTORC1 and its synergy with the AKT/GSK-3β/c-Myc axis further affect the expression of M2-related genes (71). In addition, there is also a two-way dialogue between NF-*κ*B and STAT3. The two can form a complex in the nucleus and co-activate specific genes such as Ccl2 (72); STAT3 can also induce I*κ*B expression, inhibit NF-*κ*B activity, and achieve self-limited regulation of inflammation (73). The ERK pathway is often activated in parallel with PI3 K/AKT and interacts with it at the upstream receptor level and downstream mTOR regulatory nodes (such as through TSC2) to jointly regulate cellular processes such as mRNA translation (74).

Upstream signals converge at the transcription factor level, with the KLF and IRF families playing the role of direct executors of polarization types. M2 signaling induces KLF4 through STAT6, which cooperates with STAT6 to promote M2 gene expression and suppress M1-related genes (75). IRF5, a key factor in M1 polarization, is activated by TLR-MyD88 signaling and cooperates with NF-*κ*B. Its nuclear translocation is negatively regulated by the PI3 K/AKT pathway, thereby setting the threshold for inflammatory response (76). In addition, in the type I interferon response, IRF9 forms an ISGF3 complex with STAT1/STAT2, further expanding the regulatory mechanism of signaling and transcription coupling in macrophages (77).

To sum up, exosome-mediated macrophage polarization involves a multi-level and intertwined signaling network. PI3 K/AKT-mTOR serves as a central integrated metabolism and inflammation signal, NF-*κ*B conducts a dynamic dialogue with STAT3 to regulate inflammatory processes, ERK provides collaborative input, and the KLF and IRF families are responsible for the final phenotypic output. In-depth analysis of this network is of great significance for developing precise cellular immune regulation strategies with exosomes as targets or carriers.

## 4 The exosome-macrophage axis: dual regulation in atherosclerosis

**Figure 3:**
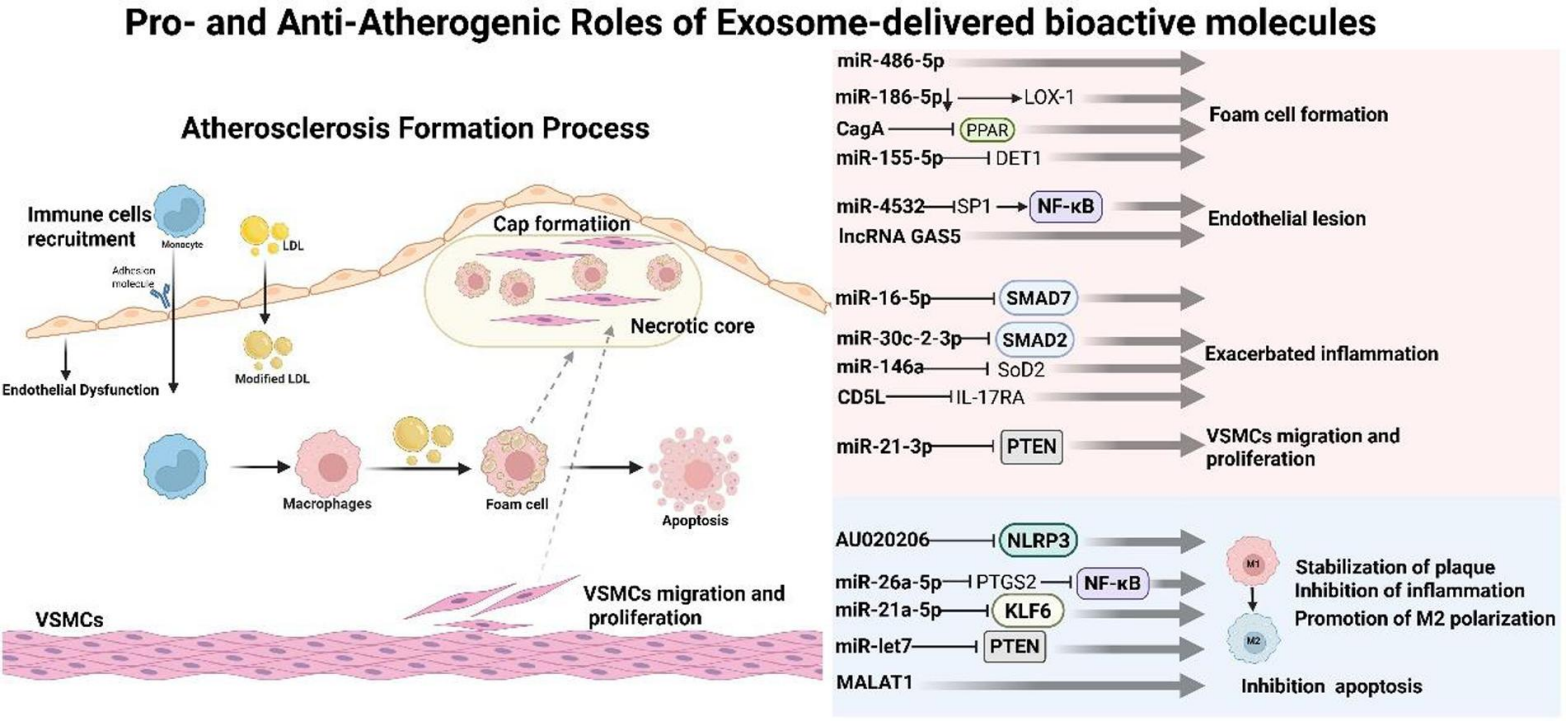
The process of atherosclerosis formation and the mechanism of promoting or inhibiting atherosclerosis by exosomes.

Cardiovascular disease, the leading cause of death worldwide, is a group of diseases of the heart and blood vessels. Atherosclerosis is considered a chronic inflammatory disease and is the beginning of many diseases affecting the carotid and coronary arteries. The formation process of atherosclerosis is complex and diverse, in which macrophages participate (Figure 3) (78). Macrophages can polarize into two different functional subgroups in response to atherosclerosis inflammatory responses: pro-inflammatory M1 and anti-inflammatory M2 (79). In the early stages of lesions, M2 macrophages are mainly present in plaques, and plaques tend to be stable. Studies have shown that M2 macrophages promote plaque stability by secreting collagen and enhancing clearance of apoptotic cells. However, as the lesion progressed, the number of M2 macrophages decreased (80), the number of M1 macrophages gradually increased, the secretion of pro-inflammatory factors increased, the plaque was easily ruptured, and the expression of the cholesterol transporter-related protein ABCA1 decreased, resulting in blocked cholesterol excretion. The resulting cholesterol accumulation perpetuates a vicious cycle by further activating macrophages and promoting their M1 polarization. Moreover, these M1 macrophages secrete matrix metalloproteinases (MMPs), including MMP2 and MMP9, which degrade the extracellular matrix within the plaque. This degradation weakens the structural integrity of the plaque, leading to instability and potential rupture, thereby elevating the risk of acute cardiovascular events (81). The distribution of M1 and M2 isoforms in different areas of AS plaques is also different; for example, M1 macrophages are more likely to infiltrate the shoulder of the plaque, and the plaque is easier to rupture (82). In the fibrous cap area, the proportions of the two subtypes are similar; protective and destructive effects are offset, thereby maintaining plaque stability (82). In advanced lesions, M1 macrophages are mainly distributed around the necrotic core of the plaque, while M2 macrophages are more common near newly formed blood vessels (83). More importantly, the M1/M2 ratio changes dynamically during the development of atherosclerosis, and subtypes can transform into each other. Khhallou-Laschet et al. conducted a polarization experiment on the body weight and found that fully polarized macrophages can repolarize into another subtype; that is to say, M1 can polarize into M2 after IL-4 induction, and M2 can polarize into M1 after LPS and IFN-*γ* induction (83). This experiment showed that polarized M1 and M2 macrophages retained their original plasticity, suggesting that the differentiation of macrophages from M1 to M2 may promote the stabilization and regression of AS plaques. Here, we summarize the latest mechanism of action of exosomes in AS.

### 4.1 Exosome-mediated pro-atherogenic effects

The occurrence and development of AS involve complex mechanisms. More and more studies have found that exosomes, as important information carriers, can regulate various processes such as macrophage foaming, inflammatory response, pyroapoptosis, and endothelial dysfunction by delivering active molecules such as functional miRNAs, proteins and lipids., forming a vicious cycle and significantly exacerbating the progression of atherosclerosis.

Exosomes derived from acute MI patients drive foam cell formation and atherosclerosis through the miR-186- 5p/LOX-1 axis: down-regulation of miR-186- 5p increases LOX-1, thereby enhancing lipid uptake and inflammatory response, a process that can be reversed by restoring miRNA expression. This study reveals the pathogenic path in which exosomes regulate the formation of macrophages and inflammatory microenvironment through miRNAs after AMI, providing a new molecular explanation for the acceleration of atherosclerosis by acute ischemic events (84). Tang et al. found that the circulating exosome miR-30c-2-3p serves as a key link between atherosclerosis and neuroinflammation in ischemic stroke. Exosome miR-30c-2-3p is significantly elevated in patients with acute ischemic stroke of the large artery atherosclerosis and can affect microglia function through the blood-brain barrier. Mechanically, the exosome miR-30c-2-3p enhances the inflammatory response of microglia by inhibiting the TGF-*β*/SMAD2 signaling pathway, exacerbating neuroinflammation and brain damage. This study reveals for the first time an exosome miRNA signaling pathway from peripheral atherosclerosis plaques to central microglia, providing a new mechanism for understanding “peripheral-central immune interaction” in stroke occurrence, and also suggests the value of miR-30c-2-3p as a potential therapeutic target or regulatory factor of neuroinflammation (85). Another study revealed that CagA-positive Helicobacter pylori deliver CagA protein through exosomes, directly exacerbating atherosclerosis. This infection significantly promotes foam cell formation and plaque progression by blocking cholesterol efflux from macrophages. Mechanically, CagA down-regulates the expression of cholesterol transporter by inhibiting the PPAR*γ*/LXR*α* signaling pathway. This study is the first time that exosome CagA is a key mediator connecting Helicobacter pylori infection and atherosclerosis (86). Chen et al. found that oxLDL stimulates macrophages to overexpress lncRNAGAS5. This molecule can be packaged by exosomes and delivered to vascular endothelial cells, inducing their apoptosis, thereby exacerbating atherosclerosis. Functional experiments have shown that knocking down GAS5 inhibits this process. This work revealed an exosome-mediated intercellular signal amplification pathway, suggesting that targeting lncRNAGAS5 has therapeutic potential (87). Under hyperglycemic conditions, macrophages release exosomes rich in miR-486-5p, exacerbating diabetic atherosclerosis by inhibiting inflammation and enhancing glycolysis (88). Studies have found that miR-155-5p is significantly over-expressed in plasma exosomes in patients with periodontitis associated atherosclerosis. Experiments have confirmed that exosome miR-155-5p drives lipid accumulation and foam cell formation through targeted inhibition of the DET1 gene (89).

Furthermore, in addition to the above-mentioned exosomes, exosomes produced by macrophages themselves can also aggravate atherosclerosis. Studies found that miR-146a was significantly enriched in serum from AS patients and exosomes derived from oxLDL-treated THP-1 cells. The exosomes can be taken up by neutrophils and induce the production of large quantities of mitochondrial ROS by targeting the expression of superoxide dismutase 2 (SOD2), thereby promoting the release of NETs and inflammatory reactions, and accelerating atherosclerosis. progress. *In vivo* experiments further showed that intravenous injection of oxLDL-treated macrophage exosomes significantly exacerbated atherosclerosis in ApoE^−/−^mice. This mechanism reveals the key role of exosomal miRNAs in macrophage-neutrophil interactions, providing a new molecular mechanism for understanding the progression of atherosclerosis (90). In a separate study, Macrophage-derived exosomes target inhibition of SMAD7 expression by delivering miR-16-5p, thereby exacerbating atherosclerosis, inflammation and oxidative stress; inhibition of miR-16- 5p can reverse this process (91). Zhu et al. found that nicotine treatment can induce macrophages to secrete miR-21-3p rich exosomes. After these exosomes are taken up by vascular smooth muscle cells (VSMC), they target PTEN gene., significantly enhance the migration and proliferation ability of VSMC, thereby accelerating the progression of atherosclerosis. *In vivo* experiments further showed that nicotine not only exacerbates lesions, but also encourages exosomes to remain in local plaques (92). In addition, the researchers found that after exosome miR-4532 is taken up by endothelial cells, it activates NF-*κ*B signaling by inhibiting transcription factor SP1, causing endothelial dysfunction and abnormal expression of inflammatory factors, which in turn recruits macrophages and promotes foam cell formation, forming a positive feedback loop (93). In another study, CD5L expression was significantly upregulated in serum and cell models of patients with AS, and was closely related to the increase in exosome marker CD63/CD81 and the increase in release of inflammatory factors (TNF-α, IL-1β, IL-6, etc.). Mechanically, CD5L is transmitted through the exosome pathway, activating IL-17RA signaling in vascular smooth muscle cells and exacerbating the inflammatory response; intervention with the exosome secretion inhibitor GW4869 or exogenous CD5L can inhibit IL-17RA expression and reduce inflammatory damage. This study shows that macrophage exosome CD5L promotes the development of AS by regulating IL-17RA-mediated inflammatory pathways, providing a basis for its use as a therapeutic target (94). To sum up, exosomes can mediate macrophages to promote AS progression through foam cell formation, inflammation amplification and other effects.

### 4.2 Exosome-mediated anti-atherogenic effects

Exosomes from different sources are equally protective in AS. Dendritic cell-derived exosomes can deliver miR-203- 3p to macrophages. By targeting cathepsin S and down-regulating the p38/MAPK signaling pathway, they inhibit the atherogenic phenotype of macrophages, effectively reducing atherosclerosis lesions (reduced foam cell formation, lipid accumulation) in mouse models and reducing disease progression (95). BMSC-EXO can specifically bind to the transcription factor CEBPB by carrying long-chain non-coding RNAAU020206, thereby inhibiting its mediated transcriptional activation of NLRP3 inflammasome, ultimately reducing macrophage catapult and atherosclerosis progression (96). Oxidized low-density lipoprotein (oxLDL)-stimulated human umbilical vein endothelial cells (HUVECs) secrete exosomes rich in long-chain non-coding RNAMALAT1, which are endocytosed by monocytes, significantly promoting their polarization towards M2-type macrophages. Inhibition of MALAT1 function reverses this polarizing effect, indicating that exosome MALAT1 is a key molecule that determines the phenotypic switching of macrophages. This discovery clarifies a new exosome-dependent communication mechanism between endothelial cells and immune cells in the development of atherosclerosis, providing a potential target for intervention in early lesions (97). He et al. revealed that QRHX regulates miR-26a-5p in macrophage-derived exosomes, targets to inhibit PTGS2 expression and block the NF-*κ*B signaling pathway, thereby promoting the polarization of macrophages from M1 to M2, ultimately reducing the inflammatory response of atherosclerosis and effectively stabilizing plaque mechanisms (50).

Many studies have shown that mesenchymal stem cells (MSCs) can treat atherosclerosis by inhibiting inflammatory responses and inhibiting plaque formation. Ma et al. found that MSC-derived exosomes double target transcription factors KLF6 and ERK1/2 signaling pathways by delivering miR-21a-5p. On the one hand, they significantly promote the polarization of macrophages towards the M2 anti-inflammatory phenotype. On the other hand, they inhibit the migration of macrophages, thereby synergistically reducing the burden of atherosclerosis plaques and inflammatory infiltration, providing a new potential strategy for AS treatment (56). Skin-derived MSCs coordinate an anti-inflammatory shift in macrophages by upregulating prostaglandin E2. This leads to a coordinated cytokine response characterized by increased IL-10 and decreased TNF-α production, effectively reprogramming the macrophage response (98). Another study reported that the exosome miR-let7, derived from mesenchymal stem cells, improved atherosclerosis in ApoE^−/−^ mice by inhibiting the HMGA2/NF-*κ*B pathway and promoted polarization of M2 macrophages in plaques. In addition, exosome miR-let7 can also decisively inhibit macrophage infiltration into plaques by inhibiting the IGF2BP1/PTEN pathway, effectively controlling the formation of atherosclerosis plaques (99).

To sum up, exosomes play an important protective role in atherosclerosis by mediating the regulation of macrophage function. Exosomes from multiple sources (such as dendritic cells, MSC, endothelial cells, etc.) can carry miRNAs by inhibiting inflammatory signaling pathways (such as NF-*κ*B, p38/MAPK, NLRP3), promoting macrophages to M2-type polarization, reducing pyrodeath and migration and other multiple mechanisms, synergistically reducing plaque formation, lipid accumulation and inflammatory responses, providing new treatment strategies and targets for AS intervention.

## 5 The exosome-macrophage axis: dual regulation in MI

MI initiates upon the rupture of an unstable atherosclerotic plaque. Following MI, the polarization state of macrophages undergoes highly dynamic and temporally regulated changes, precisely coordinating the distinct phases of cardiac repair and remodeling (100). In the early stages of MI (inflammatory phase, ∼0-3 days), resident macrophages and newly recruited Ly-6C^high^ monocytes in the infarct area are polarized into the dominant M1 phenotype driven by signals such as damage-associated molecular patterns (DAMPs) and IFN-*γ* released by necrotic cells. These cells secrete large amounts of IL-1β, TNF-α and reactive oxygen species (ROS) to clear necrotic tissue, but excessive inflammation can also expand the scope of damage (101, 102). Subsequently, the proliferation phase (∼4–14 days) was entered, and the polarization of macrophages began to undergo a critical shift towards the dominant M2 phenotype. These M2 macrophages are mainly derived from Ly-6C^low^ monocytes and inhibit inflammation, promote angiogenesis and granulation tissue formation, and lead tissue repair by secreting factors such as IL-10, TGF-*β* and VEGF (101). During the final maturation period (after ∼2 weeks and beyond), the number of macrophages generally decreases and are more concentrated in the distal myocardial area to stimulate tissue remodeling. However, if M2-type macrophages and their pro-fibrotic signals (such as TGF-*β*) continue to be over-activated at this time, they will drive fibroblasts to differentiate into muscle fibroblasts, leading to excessive deposition of extracellular matrix and myocardial fibrosis, ultimately leading to adverse ventricular remodeling and heart failure (101).

**Figure 4:**
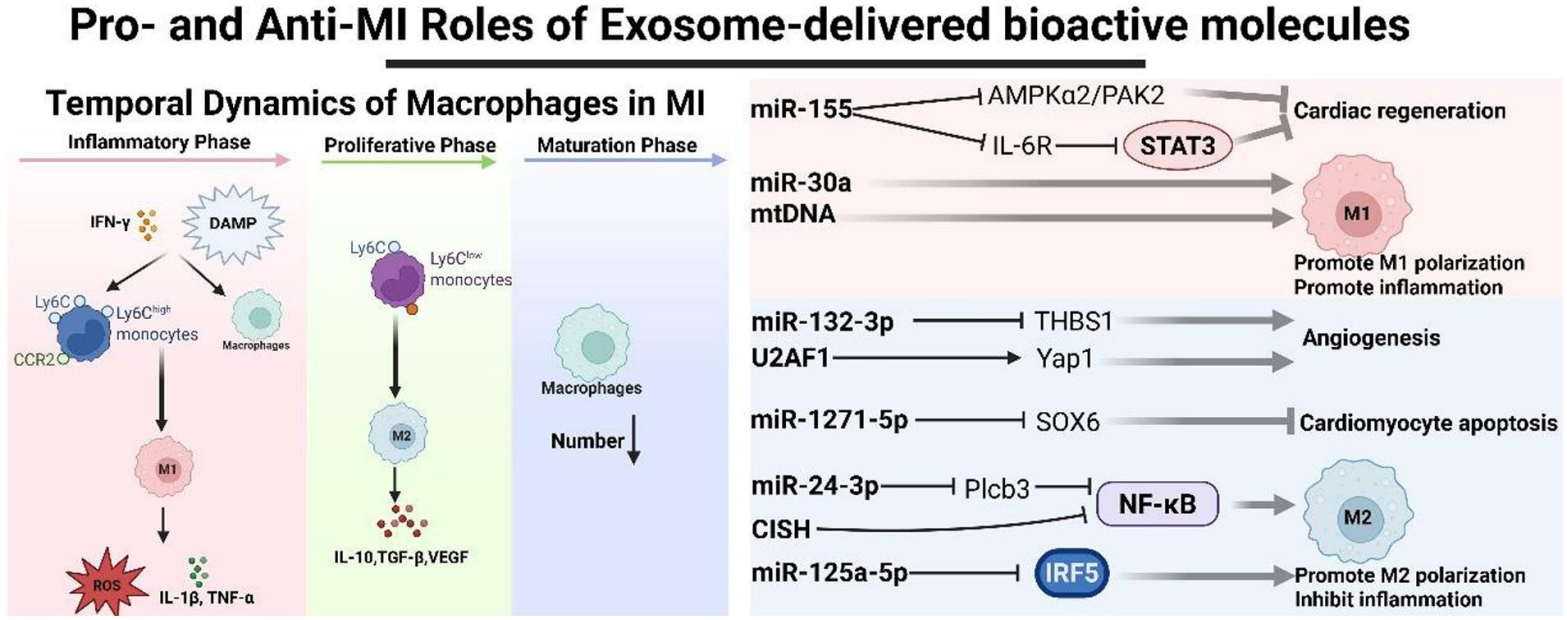
The changes of macrophages in myocardial infarction and the mechanism of exosomes promoting or inhibiting myocardial infarction.

Therefore, timely and moderate polarization of macrophages from M1 to M2 is a key determinant of functional repair of the heart. Here we summarize the relevant mechanisms by which exosomes promote and inhibit the occurrence and development of MI (Figure 4).

### 5.1 Exosome-mediated anti-MI effects

Improving MI through exosomes is feasible. Guo et al. found that M2-type macrophage-derived exosomes (M2-exos) target and inhibit the expression of THBS1 in endothelial cells by delivering miR-132- 3p, significantly promoting angiogenesis after MI, improving cardiac function and reducing the infarction size, providing a new therapeutic strategy for cardiac repair (103). In addition, exosomes from the same origin (M2-Exos) targeted inhibition of SOX6 expression by delivering miR-1271- 5p, significantly reducing hypoxia induced apoptosis in cardiomyocytes and promoting cardiac repair after acute MI (104). Recent research has found that niacinamide monophosphate stimulated macrophages regulate the alternative splicing of the Yes1-related transcription regulator (Yap1) gene by secreting exosomes rich in U2AF1 protein, thereby promoting angiogenesis and improving cardiac function after MI. In clinical studies, plasma U2AF1 levels in patients with MI were positively correlated with the quality of coronary collateral circulation, further confirming the role of U2AF1. This study not only clarifies the role of U2AF1 as a new target for promoting angiogenesis, but also provides a solid experimental and translational medicine basis for exosome-based cell-free therapy strategies (105). Human umbilical cord mesenchymal stem cell-derived exosomes (UMSC-Exos) deliver miR-24-3p to macrophages, which targets and inhibits Plcb3. This inhibition activates the downstream NF-*κ*B signaling pathway, thereby promoting a shift toward the M2 anti-inflammatory phenotype. This phenotypic change improves the inflammatory microenvironment following MI and ultimately enhances cardiac repair (106). BMSCs-Exo deliver the protein CISH to suppress the NF-*κ*B signaling pathway. This action promotes M2 macrophage polarization, thereby reducing inflammation, apoptosis, and tissue damage post-MI, and significantly improves cardiac function (107). In addition, Gong et al. found that exosomes (MSCNIC-exo) produced by mesenchymal stem cells pretreated with the cardioprotective drug nicorandil can more effectively promote the recovery of cardiac function and structural repair after AMI than ordinary exosomes (MSC-exo). The core mechanism is that miR-125a-5p rich in MSCNIC-exo can significantly drive the TRAF6/IRF5 signaling pathway in macrophages to polarize towards the M2 anti-inflammatory phenotype, thereby improving the inflammatory microenvironment in the infarction area (66). Interestingly, the researchers have developed an alginate hydrogel-based dendritic cell exosome (DEXs) delivery system (DEXs-Gel) that achieves sustained release of DEXs and significantly prolongs their residence time in the MI area. *In vivo* and *in vitro* experiments have shown that DEXs-Gel can more effectively promote the activation of regulatory T cells (Treg) and the polarization of macrophages towards the M2 repair phenotype, and increase their infiltration in the infarct border area, thereby significantly improving cardiac function (108). Literature also reports that Tregs and Treg-derived exosomes ameliorate AMI outcomes by shifting macrophages toward the M2 phenotype, thereby improving cardiac function, limiting infarct size, and reducing apoptosis. While the specific pathways are not fully elucidated, this evidence supports the concept of targeting intercellular communication for MI therapy (109).

In summary, exosome-based cell-free therapeutic strategies can reshape the cardiac immune microenvironment by delivering engineered or natural therapeutic molecules, effectively promote the polarization of macrophages towards the M2 repair phenotype, significantly inhibit inflammation, reduce cells Apoptosis and enhance angiogenesis, improve cardiac function and tissue repair.

### 5.2 Exosome-mediated pro-MI effects

More and more literature reports that exosomes can promote the progression of MI. Liu et al. study revealed a new mechanism by which M1-type macrophage-derived exosomes aggravate myocardial damage by delivering miR-155 after MI. After exosome miR-155 is taken up by endothelial cells, it can simultaneously target multiple key genes (including RAC1, PAK2, Sirt1 and AMPK*α*2), thereby inhibiting the two pro-angiogenic signaling pathways RAC1-PAK2 and Sirt1/AMPK*α*2-eNOS, significantly inhibiting angiogenesis and hindering heart repair. This discovery not only clarifies the mechanism of M1 macrophages in mediating poor myocardial repair through exosomes, but also provides a potential therapeutic target for interfering with pathological remodeling after MI (110). He et al. subsequently discovered that macrophage-derived exosomes targeted inhibition of the IL-6 receptor (IL-6R) by overexpressing and delivering miR-155 to cardiomyocytes, thereby blocking the activation of the JAK2/STAT3 signaling pathway, ultimately inhibiting cardiomyocyte proliferation and antagonizing the pro-repair effect of IL-6, thereby hindering heart regeneration after MI (111). In addition, Sun et al. revealed a new mechanism by which cardiomyocyte-derived exosomes, which cause iron death after MI, activates the Wnt/*β*-catenin signaling pathway and drives macrophages to polarize towards the pro-inflammatory M1 phenotype, thereby exacerbating cardiac damage. Studies have confirmed that MI-Exo can be internalized by macrophages, significantly upregulate the M1 marker NOS2, inhibit the expression of M2 markers IL-10 and Arg-1, and weaken the phagocytic function of macrophages; treatment with the iron death inhibitor Ferrostatin-1 can reverse these effects (112). After AMI, cardiac fibroblasts (CF) drive a macrophage-mediated inflammatory response by releasing mtExosomes rich in damaged mitochondrial components. Studies have found that mtExosomes secreted by CF treated with oxygen glucose deprivation (OGD) contain high levels of mitochondrial protein, mtDNA, mtROS and ATP, which can be effectively internalized by macrophages and promote polarization into pro-inflammatory M1 macrophages; in turn, it significantly promotes the release of pro-inflammatory factors such as IL-6 and IL-1β by activating intracellular TLRP9/NF-*κ*B/IL-6 and NLRP3/Caspase-1/IL-1β inflammatory signaling pathways. This study clarifies for the first time an intercellular communication axis composed of “ischemic CF→mtExosomes→ macrophages”, providing a new perspective for understanding immune microenvironment disorders and ventricular remodeling after AMI, and suggests that mtExosomes or their downstream inflammatory pathways can serve as potential targets for intervention in heart failure after AMI (113). Li et al. revealed a dual mechanism by which hypoxic cardiomyocytes deliver miR-30a through exosomes after acute myocardial infarction (AMI), exacerbating cardiac damage: on the one hand, when the exosome miR-30a is taken up by cardiomyocytes, it disrupts the balance of autophagy-apoptosis, which inhibits and promotes apoptosis and expands the infarction area; on the other hand, after it is internalized by cardiac macrophages, it promotes the polarization of macrophages towards the pro-inflammatory M1 phenotype, further exacerbating the inflammatory response and cardiac dysfunction. The above results suggest that exosome miR-30a can serve as a potential intervention target in AMI, and regulating its expression is expected to improve the prognosis of cardiac repair (114).

In conclusion, during MI, M1-type macrophages, iron-dead cardiomyocytes and exosomes derived from ischemic cardiac fibroblasts drive macrophages to polarize to pro-inflammatory M1 phenotype by carrying miR-155, mtDNA and other pathogenic factors, and inhibit cardiomyocyte proliferation and angiogenesis, thus aggravating inflammation, expanding infarction area and hindering tissue repair.

## 6 Exosome-mediated strategies for modulating macrophage polarization in cardiovascular diseases

### 6.1 Exosome-based therapeutic strategies targeting macrophage polarization for cardiovascular diseases

Exosomes are considered an alternative to cell therapy because they are non-living and biocompatible materials that transfer genetic cargo to recipient cells. Exosomes have huge potential in the clinical treatment of cardiovascular diseases and require more and more in-depth exploration. This review explores exosome-based strategies targeting macrophage polarization for cardiovascular disease treatment through three primary approaches: drug delivery vehicles, engineered modifications, and stem cell therapy.

Based on exosomes derived from adipose-derived stem cells (ADSCs-EXO), a delivery system was developed to load the insoluble anti-inflammatory compound icariin (ICA), forming ADSCs-EXO-ICA. This system effectively targets macrophages in the synovial tissue of joints. *In vitro* experiments confirmed that ADSCs-EXO-ICA significantly inhibits the proliferation of M1-type macrophages and promotes their conversion to the M2 phenotype (115). A hybrid exosome system (ELP), formed by fusing mesenchymal stem cell exosomes with folate-targeted liposomes, was utilized for the delivery of paclitaxel. This system demonstrated significant tumor-suppressive effects in a CT26 tumor-bearing mouse model. Mechanistic studies revealed that ELP effectively modulates the tumor immune microenvironment, with its key action being the repolarization of TAMs from the pro-tumor M2 phenotype to the anti-tumor M1 phenotype (116). Furthermore, research indicates that exosome-delivered circular RNA CBLB (circ-CBLB) can directly bind to and inhibit the TLR3/TRAF3 signaling pathway, thereby suppressing macrophage polarization towards the pro-inflammatory M1 phenotype and promoting a shift to the anti-inflammatory M2 phenotype (117). In summary, as a drug delivery platform, exosomes provide a unique solution to the challenges of precision targeting and controlled release in macrophage polarization therapy. Their intrinsic biocompatibility and modifiability enable the efficient transport of polarizing agents directly to target cells within the pathological microenvironment, offering a strategy that bridges molecular mechanisms with translational application.

Using the high plasticity of exosomes to engineer them is to improve their targeting, reduce immunogenicity, and then optimize efficacy. In order to overcome the bottleneck of short half-lives in exosomes and difficult retention of target tissues, researchers have developed a variety of engineering projects. Ge et al. prepared exosomes (CD47-Exos) by overexpressing CD47 in BMSC, and then co-loaded miR-21a using electroporation technology. CD47, acting as a “don't eat me” signal, significantly prolonged the circulation time of exosomes (still detectable 120 min after tail vein injection), while unmodified exosomes were quickly eliminated within 30 min. This collaborative strategy not only improves the stability of exosomes, but also effectively inhibits apoptosis and inflammation in animal models by delivering therapeutic miR-21a, improves cardiac function, fully demonstrating the combination of genetic engineering and drug loading application potential (118). In a study by Chen et al., IFN*γ* was efficiently loaded into exosomes derived from THP-1 macrophages, enabling precise regulation of macrophage polarization. Functional assays demonstrated that EXO-IFN*γ* effectively reprogrammed M2-type macrophages—derived from human peripheral blood mononuclear cells—into the M1 phenotype, showing significantly superior polarization-modulating ability compared to free IFN*γ* (119). In another approach, an engineered exosome delivery system was developed using mannose-modified mesenchymal stem cell exosomes to efficiently encapsulate and target miR-23b-3p to pulmonary macrophages. This system inhibited macrophage polarization toward the M1 phenotype via the miR-23b-3p/Lpar1/NF-*κ*B axis, thereby mitigating inflammatory responses (120). Taken together, these findings confirm the feasibility of modulating macrophage polarization through engineered exosomes, laying a solid foundation for their potential application in the treatment of cardiovascular diseases.

Studies have demonstrated that exosomes derived from MSCs can alleviate cardiovascular diseases such as myocardial infarction by modulating macrophage polarization. In atherosclerotic plaques of mouse models, MSC-derived exosomes significantly reduced the proportion of pro-inflammatory M1 macrophages while increasing anti-inflammatory and reparative M2 macrophages. These M2 macrophages further infiltrated the plaques, thereby suppressing local inflammation (56).Another study confirmed that exosomes promote M2 polarization of macrophages within atherosclerotic plaques of ApoE^−^/^−^ mice via the miR-let7/HMGA2/NF-*κ*B signaling pathway, attenuating disease progression (99). Research by Sun et al. showed that intravenous administration of MSC-derived exosomes regulates macrophage activation through the JAK2-STAT6 pathway, reduces the number of pro-inflammatory macrophages, and improves the cardiac inflammatory microenvironment in a diabetic cardiomyopathy mouse model, offering a potential clinical intervention strategy (121). Exosomes from adipose tissue carrying miR-196a-5p and miR-425-5p can alleviate mitochondrial dysfunction and oxidative stress in cardiomyocytes induced by ischemia, promote angiogenesis, and steer macrophages toward an M2 anti-inflammatory phenotype (122). Additionally, when delivered via MSC-derived exosomes, miR-182 specifically targets TLR4, inhibits the TLR4/NF-*κ*B pathway, and activates the PI3K/AKT pathway. This shift suppresses M1 polarization and promotes M2 polarization in a cardiac ischemia-reperfusion injury model, exerting a cardioprotective effect (123). Collectively, these findings highlight the significant therapeutic potential of stem cells, attributable to their repair and regenerative capabilities, positioning them as key candidates in cardiovascular disease treatment strategies.

This review synthesizes how exosome-based platforms—functioning as drug vehicles, engineered constructs, and stem cell-derived agents—collectively enable precise macrophage polarization control, establishing a targeted therapeutic framework for cardiovascular disease.

### 6.2 Clinical translation of exosome-based therapeutics: challenges, and prospects

Despite their considerable therapeutic promise, the clinical translation of exosome-based therapies faces fundamental challenges in standardization, manufacturing, and quality control. A primary obstacle is the lack of unified standards for production and characterization. Current isolation techniques—such as ultracentrifugation, precipitation, and size-exclusion chromatography—vary significantly in their yield, purity, and selectivity for specific vesicle subtypes. This methodological diversity leads to poorly defined product profiles and substantial batch-to-batch heterogeneity, which in turn hinders the establishment of consistent regulatory evaluation frameworks and approval pathways. Furthermore, scalable production under Good Manufacturing Practice (GMP) conditions remains a critical bottleneck. Conventional laboratory-scale methods relying on flask-based cultures and ultracentrifugation are not only low in efficiency and high in variability but are also incompatible with the demands of large-scale clinical-grade manufacturing. While scalable technologies such as hollow-fiber bioreactors are under investigation, their process development, operational control, and cost-effectiveness require further optimization. Equally important is the establishment of a rigorous quality control (QC) system, which is essential for ensuring consistent safety and efficacy. To guarantee batch-to-batch consistency, a comprehensive QC strategy spanning the entire production workflow must be implemented. This includes: (1) Process controls that standardize critical parameters such as cell source, culture conditions, and harvest timing; (2) Product characterization involving quantitative analysis of physical (e.g., hydrodynamic diameter, *ζ*-potential), biochemical (e.g., specific protein and nucleic acid markers), and functional properties of the final exosome preparation; and (3) Release testing that mandates compliance with specifications for sterility, endotoxin levels, particle concentration, and biological potency. Only through such a systematic Quality by Design (QbD) approach can therapeutic exosomes progress from bench-scale research to clinically viable and reliably effective pharmaceutical products.

Patient heterogeneity must also be considered in quality assurance. As crucial carriers of metabolic and physiological information, the composition and function of exosomes are significantly influenced by baseline patient characteristics, such as age, sex, and comorbidities. Regarding sex, fatty acid synthase levels in urinary exosomes from healthy men are reportedly higher than in women, with their positive correlation to triglycerides strengthening with age (124). In terms of comorbidities, conditions like obesity and diabetes markedly alter the circulating exosomal miRNA profile, demonstrating a progressive shift from a healthy to a disease-state signature (125). Aging has been shown to induce significant changes in the metabolomic profiles of exosomes derived from the follicular fluid of older women, affecting the reproductive microenvironment (126). This body of evidence underscores the necessity for future clinical development of exosome-based therapies to incorporate patient stratification and personalized strategies.

Furthermore, while engineering exosomes can enhance their targeting capability or drug-loading efficiency, it may also introduce new immunogenic risks (127). These risks primarily stem from: (1) residual parental cell proteins from allogeneic sources; (2) exogenously expressed engineered proteins or peptides; and (3) introduced therapeutic nucleic acids or chemical drugs. To advance clinical translation, developing effective immune evasion strategies is crucial. Primary current approaches involve surface engineering to confer “stealth” properties to exosomes. For instance, overexpression of human CD47 has been shown to effectively engage SIRP*α* on macrophages, delivering a “don't eat me” signal, thereby significantly reducing exosome clearance by the mononuclear phagocyte system and extending their circulation half-life (128). Other strategies, such as PEGylation or camouflage using native cell membranes, can also partially shield immunogenic epitopes. Additionally, using autologous cell sources for exosome production is a fundamental method to avoid immune rejection, despite challenges in achieving scalable manufacturing. Unquestionably, any therapeutic exosome product must adhere to rigorous preclinical safety assessment standards before entering clinical trials. This includes systematic evaluation of toxicity, tumorigenicity, and immunogenicity (e.g., testing for anti-drug antibodies and cytokine release responses) in relevant animal models, alongside comprehensive characterization following guidelines from the International Society for Extracellular Vesicles (ISEV) (129). Adherence to the principle of “Safety by Design” is foundational for the successful translation of engineered exosome therapies.

## 7 Conclusion and prospect

Exosomes transport functional cargo—including miRNAs, lncRNAs, and proteins—to target cells via intercellular communication, playing a crucial role in the physiological and pathological regulation of cardiovascular diseases. Central to this process is their ability to modulate macrophage polarization, which not only underscores their potential as novel diagnostic biomarkers but also reveals significant therapeutic promise. The high plasticity of macrophages provides a critical interface for exosome-based interventions. Engineering strategies such as targeted modification and drug loading have shown encouraging potential to enhance exosome-mediated macrophage reprogramming, yet their translation into cardiovascular therapies remains at an early stage. Future studies should focus on several key directions: first, to systematically elucidate how exosomal cargo beyond miRNAs—such as proteins and lncRNAs—orchestrate macrophage polarization and immune regulation; second, to develop more efficient and standardized methods for exosome isolation and characterization; third, to optimize engineering approaches that improve the targeting and polarization-modulating efficacy of exosomes; fourth, to conduct rigorous clinical trials evaluating their safety and therapeutic outcomes; and finally, to further explore their utility as biomarkers for diagnosing and prognosing cardiovascular diseases. In summary, while challenges remain, continued research into exosome-mediated macrophage polarization is poised to drive transformative advances in the diagnosis and treatment of cardiovascular disease.

## References

[1] Libby P Buring JE Badimon L Hansson GK Deanfield J Bittencourt MS Atherosclerosis. Nat Rev Dis Primers. (2019) 5:56. 10.1038/s41572-019-0106-z 31420554

[2] Murray PJ Allen JE Biswas SK Fisher EA Gilroy DW Goerdt S Macrophage activation and polarization: nomenclature and experimental guidelines. Immunity. (2014) 41:14–20. 10.1016/j.immuni.2014.06.008 25035950 PMC4123412

[3] Nahrendorf M Swirski FK. Abandoning M1/M2 for a network model of macrophage function. Circ Res. (2016) 119:414–7. 10.1161/CIRCRESAHA.116.309194 27458196 PMC4965179

[4] Kalluri R LeBleu VS. The biology, function, and biomedical applications of exosomes. Science. (2020) 367(6478). 10.1126/science.aau6977 32029601 PMC7717626

[5] Ning Y Zhou X Wang G Zhang L Wang J. Exosome miR-30a-5p regulates glomerular endothelial Cells’ EndMT and angiogenesis by modulating Notch1/VEGF signaling pathway. Curr Gene Ther. (2024) 24:159–77. 10.2174/0115665232258527230919071328 37767799

[6] Nagaram S Sen A Singh V Ps MR Dwivedi S Bansal A. Role of exosomal miRNAs and epigenetic modifications in diabetic nephropathy: insights into novel diagnostic and therapeutic strategies. Curr Gene Ther. (2025). 10.2174/0115665232376803250815065514 40873225

[7] van Niel G D'Angelo G Raposo G. Shedding light on the cell biology of extracellular vesicles. Nat Rev Mol Cell Biol. (2018) 19(4):213–28. 10.1038/nrm.2017.125 29339798

[8] Saad FA. Precision medicine: design of immune inert exosomes for targeted gene delivery. Curr Gene Ther. (2025). 10.2174/0115665232409032250908114520 41031497

[9] Marsh M van Meer G. Cell biology. No ESCRTs for exosomes. Science. (2008) 319:1191–2. 10.1126/science.1155750 18309064

[10] Zheng D Huo M Li B Wang W Piao H Wang Y The role of exosomes and exosomal MicroRNA in cardiovascular disease. Front Cell Dev Biol. (2020) 8:616161. 10.3389/fcell.2020.616161 33511124 PMC7835482

[11] Schmidt O Teis D. The ESCRT machinery. Current Biology: CB. (2012) 22:R116–20. 10.1016/j.cub.2012.01.028 22361144 PMC3314914

[12] Stuffers S Sem Wegner C Stenmark H Brech A. Multivesicular endosome biogenesis in the absence of ESCRTs. Traffic (Copenhagen, Denmark). (2009) 10(7):925–37. 10.1111/j.1600-0854.2009.00920.x 19490536

[13] Choezom D Gross JC. Neutral sphingomyelinase 2 controls exosome secretion by counteracting V-ATPase-mediated endosome acidification. J Cell Sci. (2022) 135. 10.1242/jcs.259324 35050379 PMC8919340

[14] van Niel G Charrin S Simoes S Romao M Rochin L Saftig P The tetraspanin CD63 regulates ESCRT-independent and -dependent endosomal sorting during melanogenesis. Dev Cell. (2011) 21:708–21. 10.1016/j.devcel.2011.08.019 21962903 PMC3199340

[15] Chairoungdua A Smith DL Pochard P Hull M Caplan MJ. Exosome release of *β*-catenin: a novel mechanism that antagonizes wnt signaling. J Cell Biol. (2010) 190:1079–91. 10.1083/jcb.201002049 20837771 PMC3101591

[16] Wang J Xia J Huang R Hu Y Fan J Shu Q Mesenchymal stem cell-derived extracellular vesicles alter disease outcomes via endorsement of macrophage polarization. Stem Cell Res Ther. (2020) 11:424. 10.1186/s13287-020-01937-8 32993783 PMC7522905

[17] Keshtkar S Azarpira N Ghahremani MH. Mesenchymal stem cell-derived extracellular vesicles: novel frontiers in regenerative medicine. Stem Cell Res Ther. (2018) 9:63. 10.1186/s13287-018-0791-7 29523213 PMC5845209

[18] Yu MY Jia HJ Zhang J Ran GH Liu Y Yang XH. Exosomal miRNAs-mediated macrophage polarization and its potential clinical application. Int Immunopharmacol. (2023) 117:109905. 10.1016/j.intimp.2023.109905 36848789

[19] Chen S Saeed A Liu Q Jiang Q Xu H Xiao GG Macrophages in immunoregulation and therapeutics. Signal Transduct Target Ther. (2023) 8:207. 10.1038/s41392-023-01452-1 37211559 PMC10200802

[20] Funamoto S Meili R Lee S Parry L Firtel RA. Spatial and temporal regulation of 3-phosphoinositides by PI 3-kinase and PTEN mediates chemotaxis. Cell. (2002) 109:611–23. 10.1016/S0092-8674(02)00755-9 12062104

[21] He L Chen Q Wu X. Tumour-derived exosomal miR-205 promotes ovarian cancer cell progression through M2 macrophage polarization via the PI3K/akt/mTOR pathway. J Ovarian Res. (2025) 18:28. 10.1186/s13048-025-01616-3 39955607 PMC11829414

[22] Zhang Y Chen X Li J Chen X Zhao J Liu Q Seminal plasma exosome derived miR-26-5p can regulate decidual macrophage polarization via PTEN/PI3K/AKT signaling pathway. Sci Rep. (2025) 15:9192. 10.1038/s41598-025-92880-2 40097471 PMC11914418

[23] Zhao S Mi Y Guan B Zheng B Wei P Gu Y Tumor-derived exosomal miR-934 induces macrophage M2 polarization to promote liver metastasis of colorectal cancer. J Hematol Oncol. (2020) 13:156. 10.1186/s13045-020-00991-2 33213490 PMC7678301

[24] Chen WX Wang DD Zhu B Zhu YZ Zheng L Feng ZQ Exosomal miR-222 from Adriamycin-resistant MCF-7 breast cancer cells promote macrophages M2 polarization via PTEN/akt to induce tumor progression. Aging (Albany NY). (2021) 13:10415–30. 10.18632/aging.202802 33752173 PMC8064228

[25] Shou Y Wang X Chen C Liang Y Yang C Xiao Q Exosomal miR-301a-3p from esophageal squamous cell carcinoma cells promotes angiogenesis by inducing M2 polarization of macrophages via the PTEN/PI3K/AKT signaling pathway. Cancer Cell Int. (2022) 22:153. 10.1186/s12935-022-02570-6 35436935 PMC9014619

[26] Huang Y Zhu L Li H Ye J Lin N Chen M Endometriosis derived exosomal miR-301a-3p mediates macrophage polarization via regulating PTEN-PI3K axis. Biomed Pharmacother. (2022) 147:112680. 10.1016/j.biopha.2022.112680 35124383

[27] Huang S Zhang P Yin N Xu Z Liu X Wu A Glioblastoma stem cell-derived exosomal miR-374b-3p promotes tumor angiogenesis and progression through inducing M2 macrophages polarization. iScience. (2024) 27:109270. 10.1016/j.isci.2024.109270 38487014 PMC10937837

[28] Li S Zhu X Wang X Jia J Chen M Wang Q Downregulation of exosomal miR-let-7e-5p induces macrophage M2 polarization by targeting rictor/AKT1 signal pathway in brucellosis patients. Eur J Med Res. (2025) 30:607. 10.1186/s40001-025-02867-y 40635062 PMC12239439

[29] Yuan J Hou B Guo K Zhu J Xiao H. Tumor-derived exosomal hyaluronidase 1 induced M2 macrophage polarization and promoted esophageal cancer progression. Exp Cell Res. (2024) 439:113963. 10.1016/j.yexcr.2024.113963 38382806

[30] Yang Y Wu T Wang Y Luo D Zhao Z Sun H Hypoxic tumour-derived exosomal miR-1290 exacerbates the suppression of CD8+ T cells by promoting M2 macrophage polarization. Immunology. (2024) 173:672–88. 10.1111/imm.13853 39183579

[31] Qian M Wang S Guo X Wang J Zhang Z Qiu W Hypoxic glioma-derived exosomes deliver microRNA-1246 to induce M2 macrophage polarization by targeting TERF2IP via the STAT3 and NF-*κ*B pathways. Oncogene. (2020) 39:428–42. 10.1038/s41388-019-0996-y 31485019

[32] Cai M Shi Y Zheng T Hu S Du K Ren A Mammary epithelial cell derived exosomal MiR-221 mediates M1 macrophage polarization via SOCS1/STATs to promote inflammatory response. Int Immunopharmacol. (2020) 83:106493. 10.1016/j.intimp.2020.106493 32289739

[33] Chen J Zhang K Zhi Y Wu Y Chen B Bai J Tumor-derived exosomal miR-19b-3p facilitates M2 macrophage polarization and exosomal LINC00273 secretion to promote lung adenocarcinoma metastasis via hippo pathway. Clin Transl Med. (2021) 11:e478. 10.1002/ctm2.478 34586722 PMC8435259

[34] Yan X Zhang S Jia J Yang J Song Y Duan H. Exosomal MiR-423-3p inhibits macrophage M2 polarization to suppress the malignant progression of cervical cancer. Pathol Res Pract. (2022) 235:153882. 10.1016/j.prp.2022.153882 35609397

[35] Jia H He W Wu B Zhong Z Chang Y Liu Y Cigarette smoke-induced exosomal miR-221-3p facilitates M1 macrophage polarization via the STAT3 pathway in chronic obstructive pulmonary disease. Aging (Albany NY). (2024) 16:12379–91. 10.18632/aging.206095 39213192 PMC11424577

[36] Xiao H Fu J Liu R Yan L Zhou Z Yuan J. Gastric cancer cell-derived exosomal miR-541-5p induces M2 macrophage polarization through DUSP3/JAK2/STAT3 pathway. BMC cancer. (2024) 24:957. 10.1186/s12885-024-12672-1 39103776 PMC11302208

[37] Cao W Zeng Z Sun J Chen Y Kuang F Luo S Exosome-derived circ-001422 promotes tumor-associated macrophage M2 polarization to accelerate the progression of glioma. Communications Biology. (2024) 7:1504. 10.1038/s42003-024-07134-0 39538012 PMC11561164

[38] Ye Z Yi J Jiang X Shi W Xu H Cao H Gastric cancer-derived exosomal let-7g-5p mediated by SERPINE1 promotes macrophage M2 polarization and gastric cancer progression. J Exp Clin Cancer Res. (2025) 44(1):2. 10.1186/s13046-024-03269-4 39748408 PMC11694445

[39] Sun Z Xu Y Shao B Dang P Hu S Sun H Exosomal circPOLQ promotes macrophage M2 polarization via activating IL-10/STAT3 axis in a colorectal cancer model. J Immunother Cancer. (2024) 12. 10.1136/jitc-2023-008491 PMC11116870 38782541

[40] Lin WT Wu HH Lee CW Chen YF Huang L Hui-Chun Ho J Modulation of experimental acute lung injury by exosomal miR-7704 from mesenchymal stromal cells acts through M2 macrophage polarization. Molecular therapy. Nucleic Acids. (2024) 35:102102. 10.1016/j.omtn.2023.102102 38222299 PMC10787251

[41] Zhang S Li D Wang H Liu B Du F Wang Q. CAFs-derived exosomal miR-889-3p might repress M1 macrophage polarization to boost ESCC development by regulating STAT1. Cell Biochem Biophys. (2025) 83:633–46. 10.1007/s12013-024-01496-2 39237779

[42] Lin J Lu W Cheng S Zhang Z Hu Y Chen S Exosomal CagA induces macrophage polarization and ferroptosis by JAK1-2/STAT1 signaling pathway in Helicobacter pylori-associated gastritis. Free Radical Biol Med. (2025) 239:91–103. 10.1016/j.freeradbiomed.2025.07.031 40706822

[43] Deng C Huo M Chu H Zhuang X Deng G Li W Exosome circATP8A1 induces macrophage M2 polarization by regulating the miR-1-3p/STAT6 axis to promote gastric cancer progression. Mol Cancer. (2024) 23:49. 10.1186/s12943-024-01966-4 38459596 PMC10921793

[44] Cai J Qiao B Gao N Lin N He W. Oral squamous cell carcinoma-derived exosomes promote M2 subtype macrophage polarization mediated by exosome-enclosed miR-29a-3p. American Journal of Physiology. Cell Physiology. (2019) 316:C731–40. 10.1152/ajpcell.00366.2018 30811223

[45] Jiao Y Zhang T Zhang C Ji H Tong X Xia R Exosomal miR-30d-5p of neutrophils induces M1 macrophage polarization and primes macrophage pyroptosis in sepsis-related acute lung injury. Crit Care. (2021) 25:356. 10.1186/s13054-021-03775-3 34641966 PMC8507252

[46] Lv LL Feng Y Wu M Wang B Li ZL Zhong X Exosomal miRNA-19b-3p of tubular epithelial cells promotes M1 macrophage activation in kidney injury. Cell Death Differ. (2020) 27(1):210–26. 10.1038/s41418-019-0349-y 31097789 PMC7206053

[47] Xu H Li M Pan Z Zhang Z Gao Z Zhao R miR-3184-3p enriched in cerebrospinal fluid exosomes contributes to progression of glioma and promotes M2-like macrophage polarization. Cancer Sci. (2022). 10.1111/cas.15372 PMC9357622 35411604

[48] Lyu J Sheng M Cao Y Jia L Zhang C Weng Y Ischemia and reperfusion-injured liver-derived exosomes elicit acute lung injury through miR-122-5p regulated alveolar macrophage polarization. Int Immunopharmacol. (2024) 131:111853. 10.1016/j.intimp.2024.111853 38503014

[49] Wang R Zhu Z Peng S Xu J Chen Y Wei S Exosome microRNA-125a-5p derived from epithelium promotes M1 macrophage polarization by targeting IL1RN in chronic obstructive pulmonary disease. Int Immunopharmacol. (2024) 137:112466. 10.1016/j.intimp.2024.112466 38875998

[50] He W Zhao H Xue W Luo Y Yan M Li J Qingre huoxue decoction alleviates atherosclerosis by regulating macrophage polarization through exosomal miR-26a-5p. Drug Des Devel Ther. (2024) 18:6389–411. 10.2147/DDDT.S487476 39749190 PMC11693966

[51] Ye Q Luo F Yan T. Transcription factor KLF4 regulated STAT1 to promote M1 polarization of macrophages in rheumatoid arthritis. Aging (Albany NY). (2022) 14:5669–80. 10.18632/aging.204128 35748767 PMC9365561

[52] Chu J Hu XC Li CC Li TY Fan HW Jiang GQ. KLF14 alleviated breast cancer invasion and M2 macrophages polarization through modulating SOCS3/RhoA/rock/STAT3 signaling. Cell Signal. (2022) 92:110242. 10.1016/j.cellsig.2022.110242 34998931

[53] Pan Y Hui X Hoo RLC Ye D Chan CYC Feng T Adipocyte-secreted exosomal microRNA-34a inhibits M2 macrophage polarization to promote obesity-induced adipose inflammation. J Clin Invest. (2019) 129:834–49. 10.1172/JCI123069 30667374 PMC6355214

[54] Jin X Cui L Zhao W Li X Liu L Li Y Decidualization-derived cAMP regulates phenotypic and functional conversion of decidual NK cells from CD56dimCD16− NK cells. Cell Mol Immunol. (2021) 18:1596–8. 10.1038/s41423-021-00675-y 33785840 PMC8166858

[55] Xiong J He X Xu Y Zhang W Fu F. MiR-200b is upregulated in plasma-derived exosomes and functions as an oncogene by promoting macrophage M2 polarization in ovarian cancer. J Ovarian Res. (2021) 14:74. 10.1186/s13048-021-00826-9 34078414 PMC8170822

[56] Ma J Chen L Zhu X Li Q Hu L Li H. Mesenchymal stem cell-derived exosomal miR-21a-5p promotes M2 macrophage polarization and reduces macrophage infiltration to attenuate atherosclerosis. Acta Biochim Biophys Sin (Shanghai). (2021) 53:1227–36. 10.1093/abbs/gmab102 34350954

[57] Tian S Zhou X Zhang M Cui L Li B Liu Y Mesenchymal stem cell-derived exosomes protect against liver fibrosis via delivering miR-148a to target KLF6/STAT3 pathway in macrophages. Stem Cell Res Ther. (2022) 13(1):330. 10.1186/s13287-022-03010-y 35858897 PMC9297598

[58] Yang J Yin X Zhang T. miR-124-3p derived from plasma exosomes enhances M2 macrophage polarization to treat acute lung injury. J Immunol. (2025). 10.1093/jimmun/vkaf097 40619875

[59] Zhao W Wu Y Wang Y Li T Liu Q Hou Z. Exosomal miR-92a-3p modulates M2 macrophage polarization in colorectal cancer: implications for tumor migration and angiogenesis. Medical Oncology (Northwood, London, England). (2025) 42:96. 10.1007/s12032-025-02635-2 40059261

[60] Hu Y Li Y Xiong H Zhang Y Wang F Zhuo W Exosomal SLC16A1-AS1-induced M2 macrophages polarization facilitates hepatocellular carcinoma progression. Int J Biol Sci. (2024) 20(11):4341–63. 10.7150/ijbs.94440 39247822 PMC11379075

[61] Qian W Wu E Chen H Yao J Wang J Zhou Y MSCs-exosomes can promote macrophage M2 polarization via exosomal miR-21-5p through mesenteric injection: a promising way to attenuate murine colitis. Journal of Crohn’s & Colitis. (2024). 10.1093/ecco-jcc/jjae110 39001689

[62] Qin X Niu R Tan Y Huang Y Ren W Zhou W Exosomal PSM-E inhibits macrophage M2 polarization to suppress prostate cancer metastasis through the RACK1 signaling axis. Biomark Res. (2024) 12:138. 10.1186/s40364-024-00685-8 39538297 PMC11562865

[63] Thompson CD Matta B Barnes BJ. Therapeutic targeting of IRFs: pathway-dependence or structure-based? Front Immunol. (2018) 9:2622. 10.3389/fimmu.2018.02622 30515152 PMC6255967

[64] Li Z Li Q Tong K Zhu J Wang H Chen B BMSC-derived exosomes promote tendon-bone healing after anterior cruciate ligament reconstruction by regulating M1/M2 macrophage polarization in rats. Stem Cell Res Ther. (2022) 13:295. 10.1186/s13287-022-02975-0 35841008 PMC9284827

[65] Chang Q Hao Y Wang Y Zhou Y Zhuo H Zhao G. Bone marrow mesenchymal stem cell-derived exosomal microRNA-125a promotes M2 macrophage polarization in spinal cord injury by downregulating IRF5. Brain Res Bull. (2021) 170:199–210. 10.1016/j.brainresbull.2021.02.015 33609602

[66] Gong ZT Xiong YY Ning Y Tang RJ Xu JY Jiang WY Nicorandil-pretreated mesenchymal stem cell-derived exosomes facilitate cardiac repair after myocardial infarction via promoting macrophage M2 polarization by targeting miR-125a-5p/TRAF6/IRF5 signaling pathway. Int J Nanomed. (2024) 19:2005–24. 10.2147/IJN.S441307 PMC10926597 38469055

[67] Wang L Shen K Gao Z Ren M Wei C Yang Y Melanoma derived exosomes amplify radiotherapy induced abscopal effect via IRF7/I-IFN axis in macrophages. Adv Sci (Weinh). (2024) 11:e2304991. 10.1002/advs.202304991 38286661 PMC10987102

[68] Ren J Lei G Dong A Cao S Han X Li H. Therapeutic potential of ADSC-derived exosomes in acute lung injury by regulating macrophage polarization through IRF7/NLRP3 signaling. Int Immunopharmacol. (2025) 156:114658. 10.1016/j.intimp.2025.114658 40252464

[69] Dan HC Cooper MJ Cogswell PC Duncan JA Ting JP Baldwin AS. Akt-dependent regulation of NF-{kappa}B is controlled by mTOR and raptor in association with IKK. Genes Dev. (2008) 22:1490–500. 10.1101/gad.1662308 18519641 PMC2418585

[70] Lee DF Kuo HP Chen CT Hsu JM Chou CK Wei Y IKK beta suppression of TSC1 links inflammation and tumor angiogenesis via the mTOR pathway. Cell. (2007) 130:440–55. 10.1016/j.cell.2007.05.058 17693255

[71] Shen Y Bao R Ye X Li H Sun Y Ren Q Morinda officinalis iridoid glycosides, as an inhibitor of GSK-3β, alleviates rheumatoid arthritis through inhibition of NF-*κ*B and JAK2/STAT3 pathway. Front Pharmacol. (2024) 15:1435274. 10.3389/fphar.2024.1435274 39444614 PMC11496184

[72] Yu H Pardoll D Jove R. STATs in cancer inflammation and immunity: a leading role for STAT3. Nat Rev Cancer. (2009) 9:798–809. 10.1038/nrc2734 19851315 PMC4856025

[73] Zhang H Hu H Greeley N Jin J Matthews AJ Ohashi E STAT3 Restrains RANK- and TLR4-mediated signalling by suppressing expression of the E2 ubiquitin-conjugating enzyme Ubc13. Nat Commun. (2014) 5:5798. 10.1038/ncomms6798 25503582 PMC4270087

[74] Ma L Chen Z Erdjument-Bromage H Tempst P Pandolfi PP. Phosphorylation and functional inactivation of TSC2 by erk implications for tuberous sclerosis and cancer pathogenesis. Cell. (2005) 121:179–93. 10.1016/j.cell.2005.02.031 15851026

[75] Liao X Sharma N Kapadia F Zhou G Lu Y Hong H Krüppel-like factor 4 regulates macrophage polarization. J Clin Invest. (2011) 121:2736–49. 10.1172/JCI45444 21670502 PMC3223832

[76] Ban T Sato GR Nishiyama A Akiyama A Takasuna M Umehara M Lyn kinase suppresses the transcriptional activity of IRF5 in the TLR-MyD88 pathway to restrain the development of autoimmunity. Immunity. (2016) 45:319–32. 10.1016/j.immuni.2016.07.015 27521268

[77] Platanitis E Decker T. Regulatory networks involving STATs, IRFs, and NF*κ*B in inflammation. Front Immunol. (2018) 9:2542. 10.3389/fimmu.2018.02542 30483250 PMC6242948

[78] Yang K Xiao Q Niu M Pan X Zhu X. Exosomes in atherosclerosis: convergence on macrophages. Int J Biol Sci. (2022) 18:3266–81. 10.7150/ijbs.71862 35637946 PMC9134907

[79] Zhao J Ling L Zhu W Ying T Yu T Sun M M1/M2 re-polarization of kaempferol biomimetic NPs in anti-inflammatory therapy of atherosclerosis. J Control Release. (2023) 353:1068–83. 10.1016/j.jconrel.2022.12.041 36549391

[80] Bisgaard LS Mogensen CK Rosendahl A Cucak H Nielsen LB Rasmussen SE Bone marrow-derived and peritoneal macrophages have different inflammatory response to oxLDL and M1/M2 marker expression - implications for atherosclerosis research. Sci Rep. (2016) 6:35234. 10.1038/srep35234 27734926 PMC5062347

[81] Seifert R Kuhlmann MT Eligehausen S Kiefer F Hermann S Schäfers M. Molecular imaging of MMP activity discriminates unstable from stable plaque phenotypes in shear-stress induced murine atherosclerosis. PLoS One. (2018) 13:e0204305. 10.1371/journal.pone.0204305 30304051 PMC6179381

[82] Stöger JL Gijbels MJ van der Velden S Manca M van der Loos CM Biessen EA Distribution of macrophage polarization markers in human atherosclerosis. Atherosclerosis. (2012) 225:461–8. 10.1016/j.atherosclerosis.2012.09.013 23078881

[83] Khallou-Laschet J Varthaman A Fornasa G Compain C Gaston AT Clement M Macrophage plasticity in experimental atherosclerosis. PLoS One. (2010) 5:e8852. 10.1371/journal.pone.0008852 20111605 PMC2810335

[84] Ding J Li H Liu W Wang X Feng Y Guan H miR-186-5p dysregulation in serum exosomes from patients with AMI aggravates atherosclerosis via targeting LOX-1. Int J Nanomed. (2022) 17:6301–16. 10.2147/IJN.S383904 PMC9758944 36536941

[85] Tang Y Dong MH Pang XW Zhang H Chu YH Zhou LQ Macrophage exosomal miR-30c-2-3p in atherosclerotic plaques aggravates microglial neuroinflammation during large-artery atherosclerotic stroke via TGF-*β*/SMAD2 pathway. J Neuroinflammation. (2024) 21:292. 10.1186/s12974-024-03281-7 39511683 PMC11545805

[86] Yang S Xia YP Luo XY Chen SL Li BW Ye ZM Exosomal CagA derived from helicobacter pylori-infected gastric epithelial cells induces macrophage foam cell formation and promotes atherosclerosis. J Mol Cell Cardiol. (2019) 135:40–51. 10.1016/j.yjmcc.2019.07.011 31352044

[87] Chen L Yang W Guo Y Chen W Zheng P Zeng J Exosomal lncRNA GAS5 regulates the apoptosis of macrophages and vascular endothelial cells in atherosclerosis. PLoS One. (2017) 12:e0185406. 28945793 10.1371/journal.pone.0185406 PMC5612752

[88] Bouchareychas L Duong P Phu TA Alsop E Meechoovet B Reiman R High glucose macrophage exosomes enhance atherosclerosis by driving cellular proliferation & hematopoiesis. iScience. (2021) 24(8):102847. 10.1016/j.isci.2021.102847 34381972 PMC8333149

[89] Yang WW Li QX Wang F Zhang XR Zhang XL Wang M Exosomal miR-155-5p facilitates lipopolysaccharide transport and foam cell formation: a novel link between periodontitis and atherosclerosis. J Periodontal Res. (2024). 10.1111/jre.13369 39604303

[90] Zhang YG Song Y Guo XL Miao RY Fu YQ Miao CF Exosomes derived from oxLDL-stimulated macrophages induce neutrophil extracellular traps to drive atherosclerosis. Cell Cycle (Georgetown, Tex.). (2019) 18:2674–84. 10.1080/15384101.2019.1654797 31416388 PMC6773244

[91] Chen F Li J She J Chen T Yuan Z. Exosomal microRNA-16-5p from macrophage exacerbates atherosclerosis via modulating mothers against decapentaplegic homolog 7. Microvasc Res. (2022) 142:104368. 10.1016/j.mvr.2022.104368 35378135

[92] Zhu J Liu B Wang Z Wang D Ni H Zhang L Exosomes from nicotine-stimulated macrophages accelerate atherosclerosis through miR-21-3p/PTEN-mediated VSMC migration and proliferation. Theranostics. (2019) 9:6901–19. 10.7150/thno.37357 31660076 PMC6815950

[93] Liu P Wang S Wang G Zhao M Du F Li K Macrophage-derived exosomal miR-4532 promotes endothelial cells injury by targeting SP1 and NF-*κ*B P65 signalling activation. J Cell Mol Med. (2022) 26:5165–80. 10.1111/jcmm.17541 36071548 PMC9575109

[94] Wang L Liu L Qian W Zheng Z. CD5L secreted by macrophage on atherosclerosis progression based on lipid metabolism induced inflammatory damage. Arch Immunol Ther Exp. (2022) 70:10. 10.1007/s00005-022-00643-y 35249136

[95] Lin B Xie W Zeng C Wu X Chen A Li H Transfer of exosomal microRNA-203-3p from dendritic cells to bone marrow-derived macrophages reduces development of atherosclerosis by downregulating ctss in mice. Aging (Albany NY). (2021) 13:15638–58. 10.18632/aging.103842 34077394 PMC8221304

[96] Zhang N Luo Y Shao J Sun H Ma K Gao X. Exosomal long non-coding RNA AU020206 alleviates macrophage pyroptosis in atherosclerosis by suppressing CEBPB-mediated NLRP3 transcription. Exp Cell Res. (2024) 438:114054. 10.1016/j.yexcr.2024.114054 38657723

[97] Huang C Han J Wu Y Li S Wang Q Lin W Exosomal MALAT1 derived from oxidized low-density lipoprotein-treated endothelial cells promotes M2 macrophage polarization. Mol Med Rep. (2018) 18:509–15. 29750307 10.3892/mmr.2018.8982

[98] Li Q Sun W Wang X Zhang K Xi W Gao P. Skin-derived mesenchymal stem cells alleviate atherosclerosis via modulating macrophage function. Stem Cells Transl Med. (2015) 4:1294–301. 10.5966/sctm.2015-0020 26400926 PMC4622403

[99] Li J Xue H Li T Chu X Xin D Xiong Y Exosomes derived from mesenchymal stem cells attenuate the progression of atherosclerosis in ApoE(-/-) mice via miR-let7 mediated infiltration and polarization of M2 macrophage. Biochem Biophys Res Commun. (2019) 510:565–72. 10.1016/j.bbrc.2019.02.005 30739785

[100] Frangogiannis NG. The inflammatory response in myocardial injury, repair, and remodelling. Nat Rev Cardiol. (2014) 11:255–65. 10.1038/nrcardio.2014.28 24663091 PMC4407144

[101] Peet C Ivetic A Bromage DI Shah AM. Cardiac monocytes and macrophages after myocardial infarction. Cardiovasc Res. (2020) 116:1101–12. 10.1093/cvr/cvz336 31841135 PMC7177720

[102] Hilgendorf I Gerhardt LM Tan TC Winter C Holderried TA Chousterman BG Ly-6Chigh monocytes depend on Nr4a1 to balance both inflammatory and reparative phases in the infarcted myocardium. Circ Res. (2014) 114:1611–22. 10.1161/CIRCRESAHA.114.303204 24625784 PMC4017349

[103] Guo H Li Z Xiao B Huang R. M2 macrophage-derived exosomes promote angiogenesis and improve cardiac function after myocardial infarction. Biol Direct. (2024) 19:43. 10.1186/s13062-024-00485-y 38840223 PMC11155164

[104] Long R Gao L Li Y Li G Qin P Wei Z M2 macrophage-derived exosomes carry miR-1271-5p to alleviate cardiac injury in acute myocardial infarction through down-regulating SOX6. Mol Immunol. (2021) 136:26–35. 10.1016/j.molimm.2021.05.006 34058620

[105] Gong M Li H Jiao L Liu T Zhang Y Liu J Macrophage-secreted U2AF1 orchestrates coronary artery angiogenesis to facilitate myocardial infarction repair through the regulation of Yap1 Variable splicing. Engineering. (2025) 50:203–19. 10.1016/j.eng.2025.06.006

[106] Zhu F Chen Y Li J Yang Z Lin Y Jiang B Human umbilical cord mesenchymal stem cell-derived exosomes attenuate myocardial infarction injury via miR-24-3p-promoted M2 macrophage polarization. Advanced Biology. (2022) 6:e2200074. 10.1002/adbi.202200074 35818695

[107] Ouyang M Yang Y Yu G Zhao J Peng Y. BMSCs-derived exosome CISH alleviates myocardial infarction by inactivating the NF-*κ*B pathway to stimulate macrophage M2 polarization. Cardiovasc Toxicol. (2024) 24:422–34. 10.1007/s12012-024-09847-4 38512651

[108] Zhang Y Cai Z Shen Y Lu Q Gao W Zhong X Hydrogel-load exosomes derived from dendritic cells improve cardiac function via treg cells and the polarization of macrophages following myocardial infarction. J Nanobiotechnology. (2021) 19:271. 10.1186/s12951-021-01016-x 34496871 PMC8424987

[109] Hu H Wu J Cao C Ma L. Exosomes derived from regulatory T cells ameliorate acute myocardial infarction by promoting macrophage M2 polarization. IUBMB life. (2020) 72:2409–19. 10.1002/iub.2364 32842172

[110] Liu S Chen J Shi J Zhou W Wang L Fang W M1-like macrophage-derived exosomes suppress angiogenesis and exacerbate cardiac dysfunction in a myocardial infarction microenvironment. Basic Res Cardiol. (2020) 115:22. 10.1007/s00395-020-0781-7 32112145

[111] He X Liu S Zhang Z Liu Q Dong J Lin Z M1 macrophage-derived exosomes inhibit cardiomyocyte proliferation through delivering miR-155. BMC Cardiovasc Disord. (2024) 24:365. 10.1186/s12872-024-03893-0 39014329 PMC11251235

[112] Sun S Wu Y Maimaitijiang A Huang Q Chen Q. Ferroptotic cardiomyocyte-derived exosomes promote cardiac macrophage M1 polarization during myocardial infarction. PeerJ. (2022) 10:e13717. 10.7717/peerj.13717 35818358 PMC9270880

[113] Zhao Y Chen L Ma G. Cardiac fibroblasts-mtExosomes-macrophages axis aggravates ventricular remodeling after acute myocardial infarction. Eur Heart J. (2023) 44. 10.1093/eurheartj/ehad655.3068

[114] Li YY Chen HR Yang Y Pan YJ Yuan QC Liu YZ. Murine exosomal miR-30a aggravates cardiac function after acute myocardial infarction via regulating cell fate of cardiomyocytes and cardiac resident macrophages. Int J Cardiol. (2024) 414:132395. 10.1016/j.ijcard.2024.132395 39074620

[115] Yan Q Liu H Sun S Yang Y Fan D Yang Y Adipose-derived stem cell exosomes loaded with icariin alleviates rheumatoid arthritis by modulating macrophage polarization in rats. J Nanobiotechnology. (2024) 22(1):423. 10.1186/s12951-024-02711-1 39026367 PMC11256651

[116] Wang X Li D Li G Chen J Yang Y Bian L Enhanced therapeutic potential of hybrid exosomes loaded with paclitaxel for cancer therapy. Int J Mol Sci. (2024) 25. 10.3390/ijms25073645 PMC11012016 38612457

[117] Zhang M Wan L Zhang X Wang S Li F Yan D. Exosome circ-CBLB promotes M1 macrophage polarization in rheumatoid arthritis through the TLR3/TRAF3 signaling axis. Front Immunol. (2025) 16:1627389. 10.3389/fimmu.2025.1627389 40746530 PMC12310475

[118] Wei Z Chen Z Zhao Y Fan F Xiong W Song S Mononuclear phagocyte system blockade using extracellular vesicles modified with CD47 on membrane surface for myocardial infarction reperfusion injury treatment. Biomaterials. (2021) 275:121000. 10.1016/j.biomaterials.2021.121000 34218049

[119] Chen Y Du Z Jiang W Kong L Huang D Zeng C Engineered IFN*γ*-loaded exosomes reprogram macrophage polarization and suppress tumor cell proliferation *in vitro*. Int Immunopharmacol. (2025) 162:115170. 10.1016/j.intimp.2025.115170 40639052

[120] Lin J Yang L Liu T Zhao H Liu Y Shu F Mannose-modified exosomes loaded with MiR-23b-3p target alveolar macrophages to alleviate acute lung injury in sepsis. J Controlled Release. (2025) 379:832–47. 10.1016/j.jconrel.2025.01.073 39870316

[121] Sun X Shan A Wei Z Xu B. Intravenous mesenchymal stem cell-derived exosomes ameliorate myocardial inflammation in the dilated cardiomyopathy. Biochem Biophys Res Commun. (2018) 503:2611–8. 10.1016/j.bbrc.2018.08.012 30126637

[122] de Almeida Oliveira NC Neri EA Silva CM Valadão IC Fonseca-Alaniz MH Zogbi C Multicellular regulation of miR-196a-5p and miR-425-5 from adipose stem cell-derived exosomes and cardiac repair. Clinical Science (London, England: 1979). (2022) 136:1281–301. 10.1042/CS20220216 35894060

[123] Zhao J Li X Hu J Chen F Qiao S Sun X Mesenchymal stromal cell-derived exosomes attenuate myocardial ischaemia-reperfusion injury through miR-182-regulated macrophage polarization. Cardiovasc Res. (2019) 115:1205–16. 10.1093/cvr/cvz040 30753344 PMC6529919

[124] Li T Meng W Liu TC Wang YZ Zhang M. Sex differences in FASN protein concentrations in urinary exosomes related to serum triglycerides levels in healthy adults. Lipids Health Dis. (2023) 22(1):176. 10.1186/s12944-023-01936-7 37858194 PMC10588030

[125] Kim H Bae YU Lee H Kim H Jeon JS Noh H Effect of diabetes on exosomal miRNA profile in patients with obesity. BMJ Open Diabetes Res Care. (2020) 8. 10.1136/bmjdrc-2020-001403 PMC7473624 32883688

[126] Gu Y Zhang X Wang R Wei Y Peng H Wang K Metabolomic profiling of exosomes reveals age-related changes in ovarian follicular fluid. Eur J Med Res. (2024) 29:4. 10.1186/s40001-023-01586-6 38173013 PMC10762974

[127] Ma X Liu B Fan L Liu Y Zhao Y Ren T Native and engineered exosomes for inflammatory disease. Nano Res. (2023) 16:6991–7006. 10.1007/s12274-022-5275-5 36591564 PMC9793369

[128] Lin YK Pan YF Jiang TY Chen YB Shang TY Xu MY Blocking the SIRP*α*-CD47 axis promotes macrophage phagocytosis of exosomes derived from visceral adipose tissue and improves inflammation and metabolism in mice. J Biomed Sci. (2025) 32:31. 10.1186/s12929-025-01124-y 40016734 PMC11869713

[129] Rayyan M Zheutlin A Byrd JB. Clinical research using extracellular vesicles: insights from the international society for extracellular vesicles 2018 annual meeting. J Extracell Vesicles. (2018) 7:1535744. 10.1080/20013078.2018.1535744 31162489 PMC6211232

